# Supplementary figures and images for: Inhibition of GSK3α,β rescues cognitive phenotypes in a preclinical mouse model of CTNNB1 syndrome
Source: EMBO Mol Med. 2024 Aug 5;16(9):8. doi: 10.1038/s44321-024-00110-5 (PMC11393422; doi:10.1038/s44321-024-00110-5)

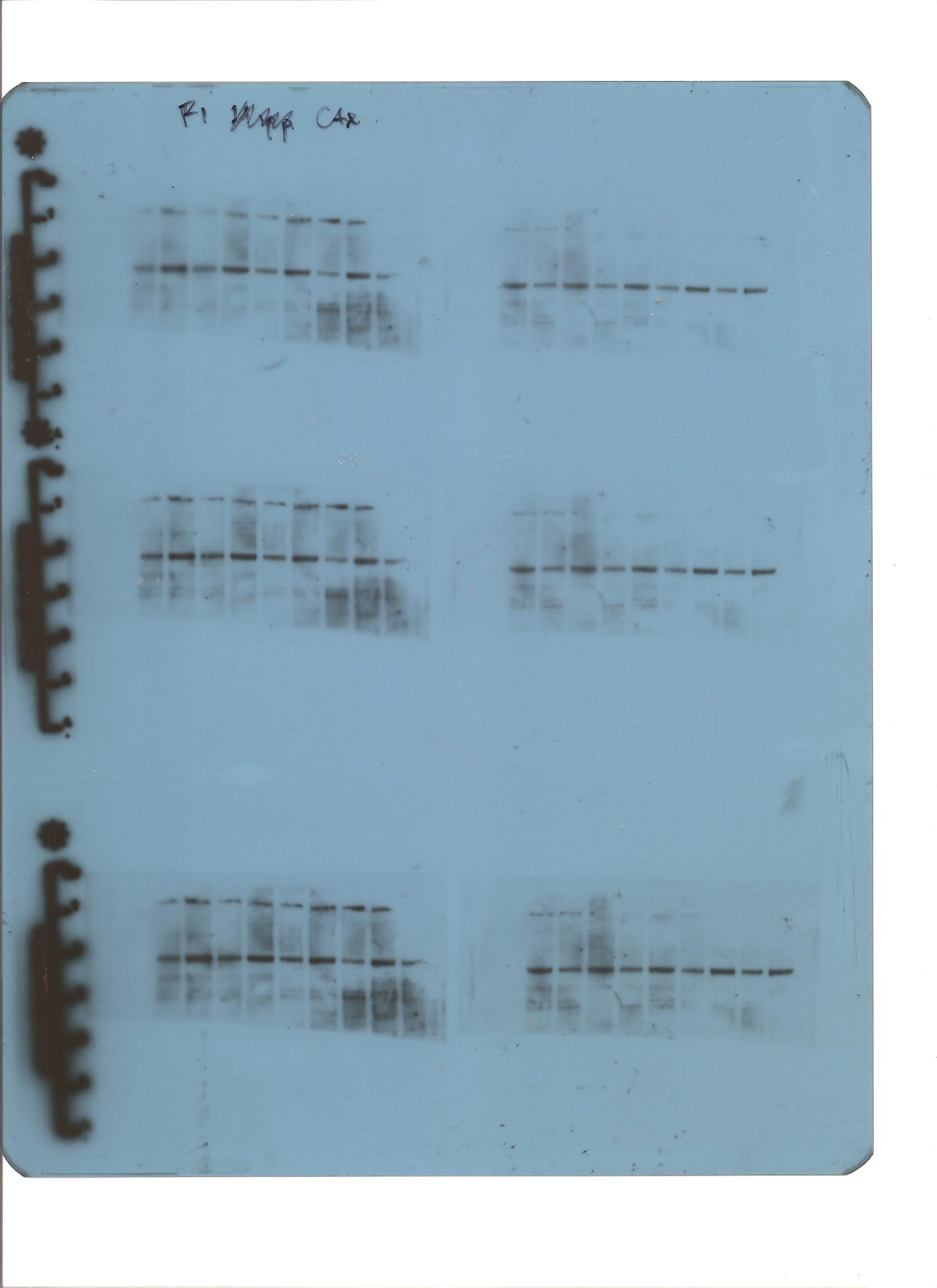

Supplement: Supplementary file 2 — Source data Fig. 1 [file 44321_2024_110_MOESM2_ESM.zip › 1C/1C Ctx Bcat.jpg]

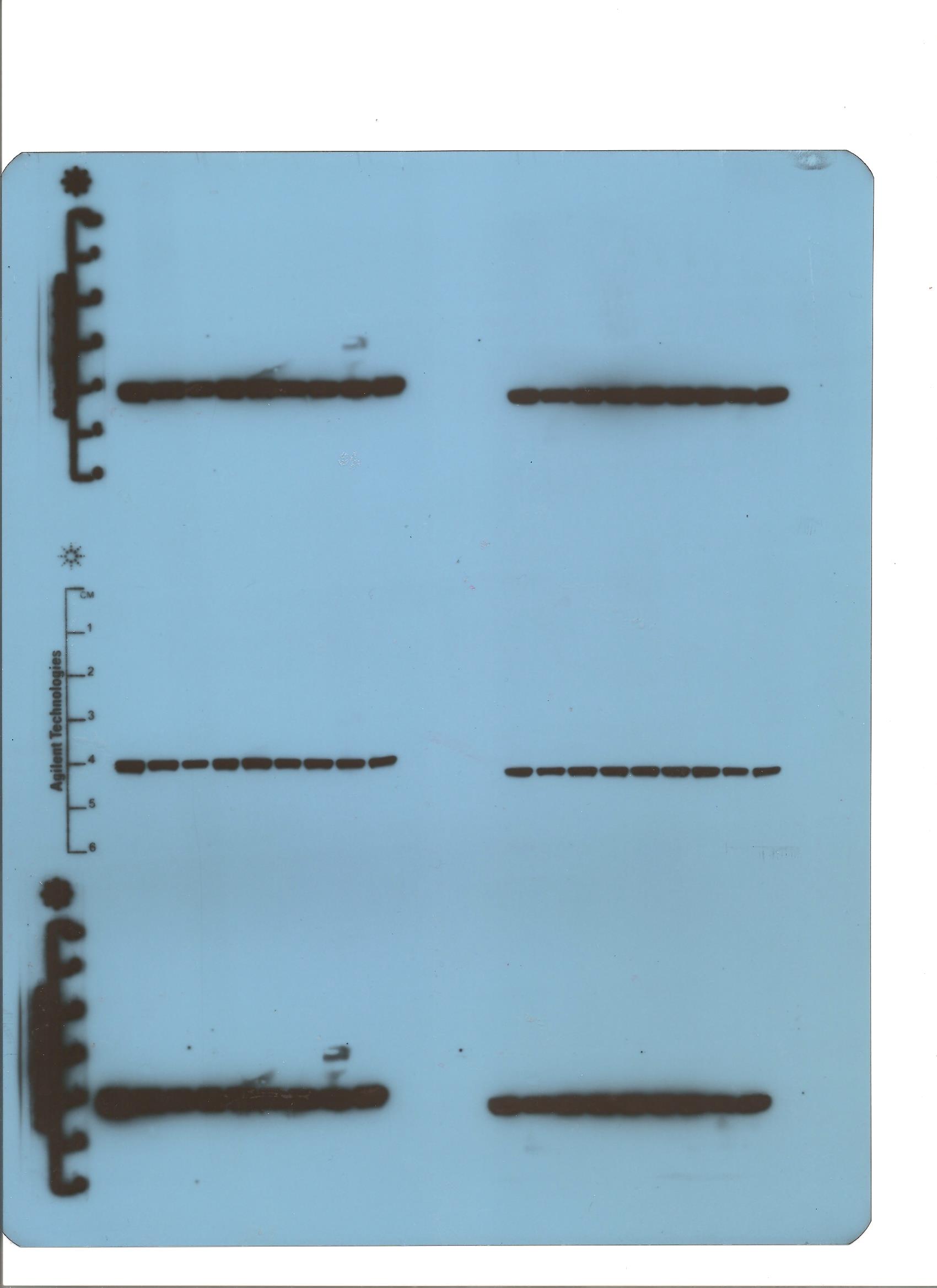

Supplement: Supplementary file 2 — Source data Fig. 1 [file 44321_2024_110_MOESM2_ESM.zip › 1C/1C Ctx GAPDH.jpg]

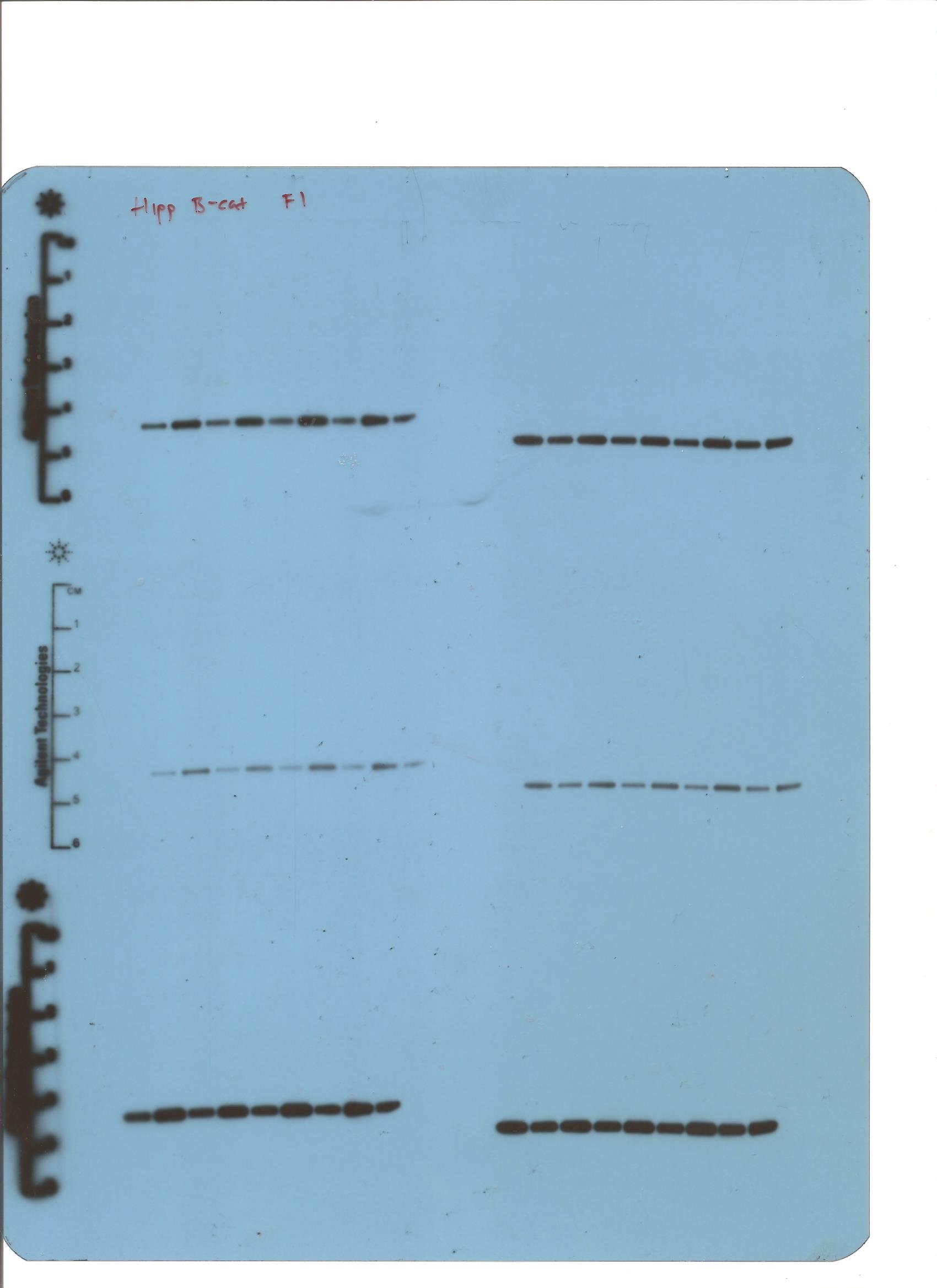

Supplement: Supplementary file 2 — Source data Fig. 1 [file 44321_2024_110_MOESM2_ESM.zip › 1C/1C Hipp Bcat.jpg]

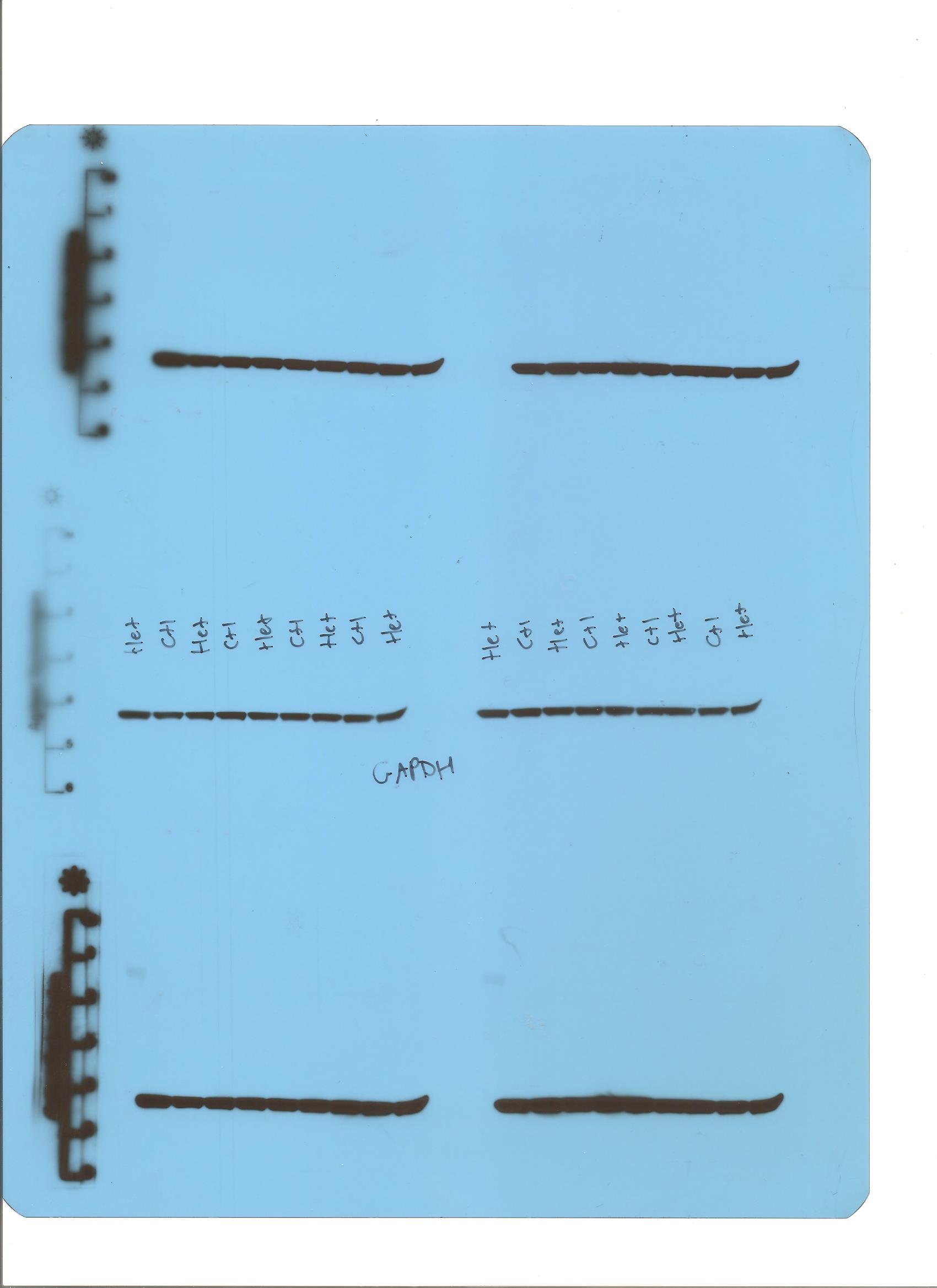

Supplement: Supplementary file 2 — Source data Fig. 1 [file 44321_2024_110_MOESM2_ESM.zip › 1C/1C Hipp GAPDH.jpg]

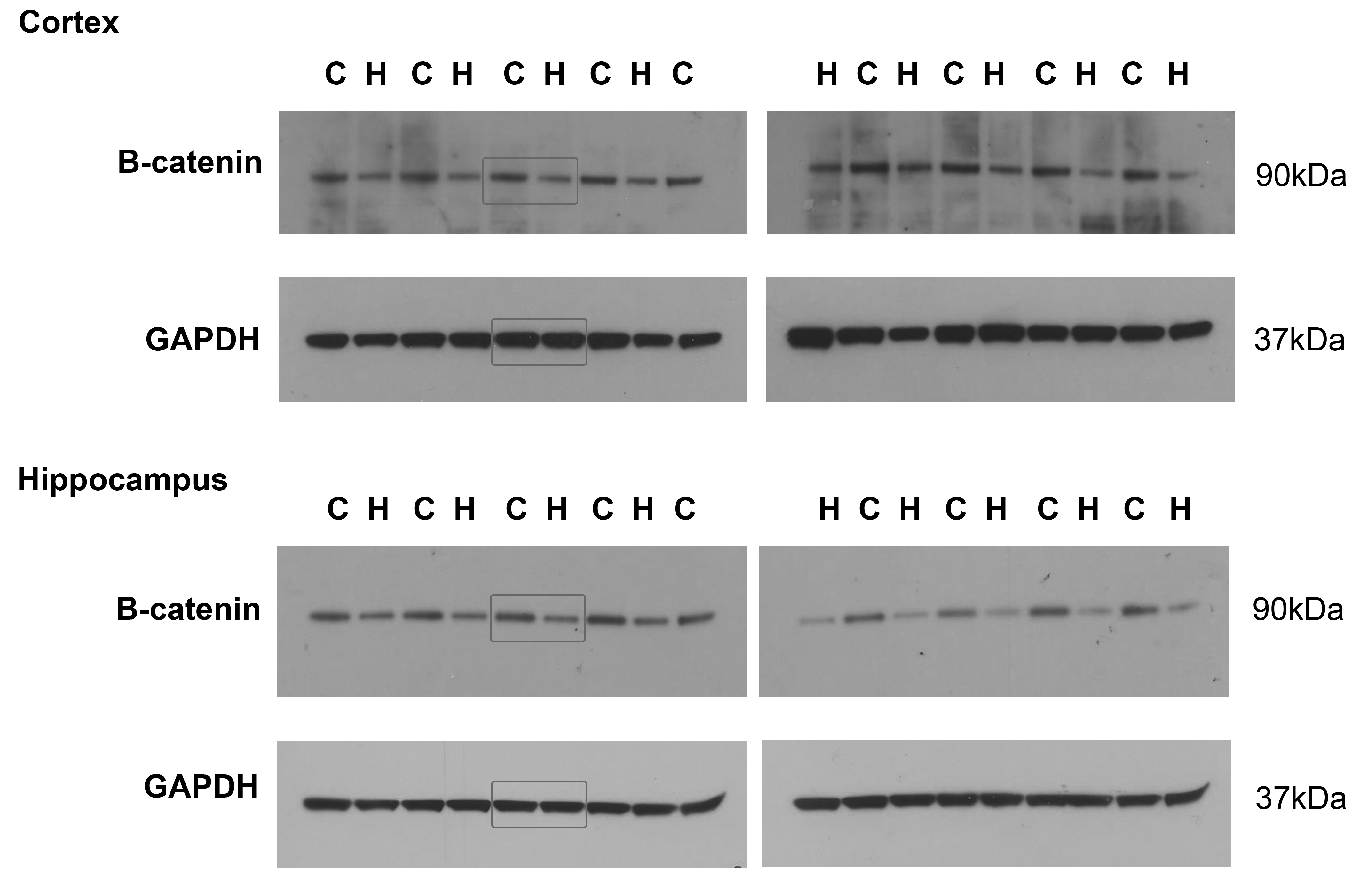

Supplement: Supplementary file 2 — Source data Fig. 1 [file 44321_2024_110_MOESM2_ESM.zip › 1C/Figure 1C Blots Annotated.tif]

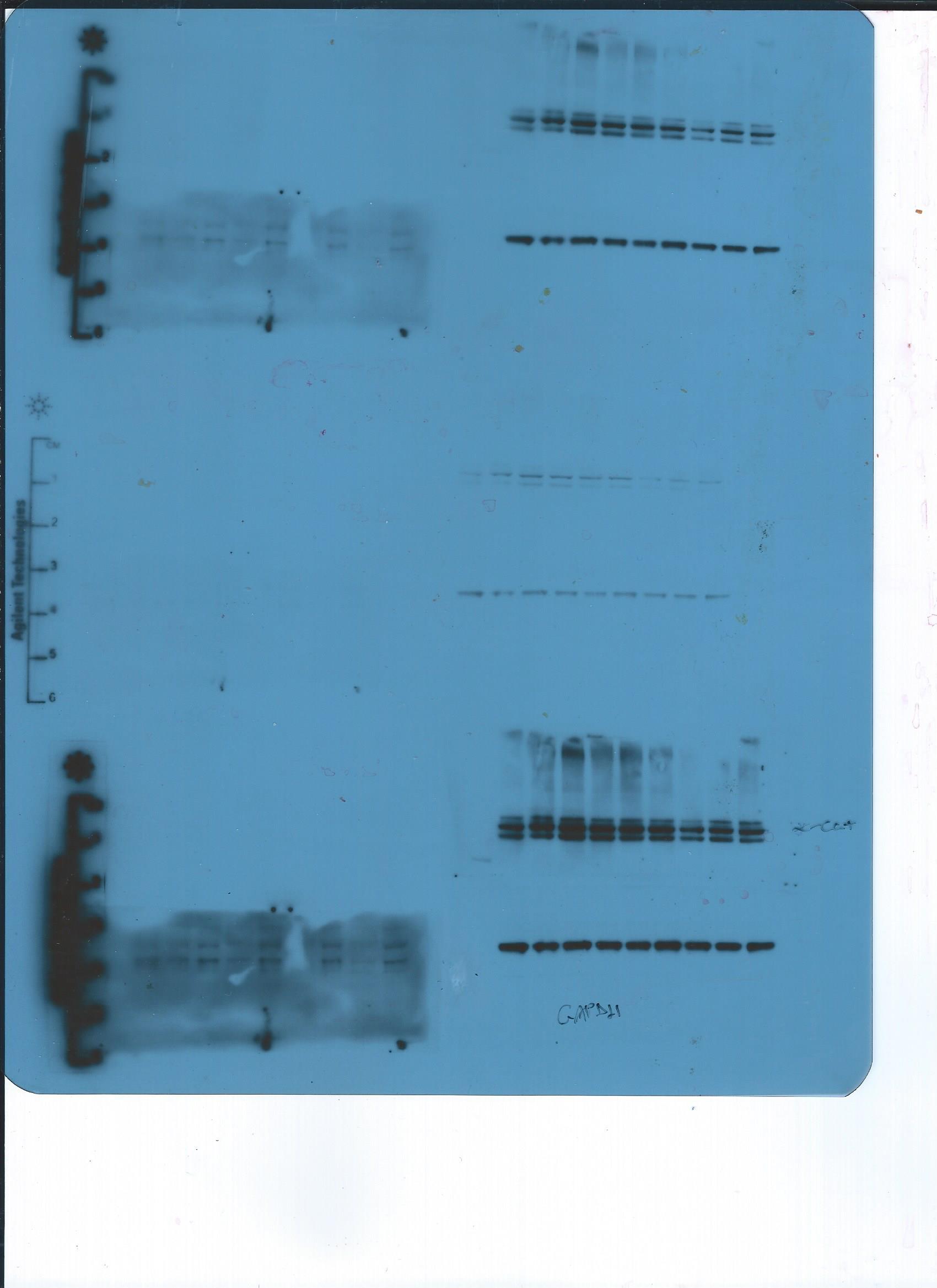

Supplement: Supplementary file 4 — Source data Fig. 3 [file 44321_2024_110_MOESM4_ESM.zip › 3A/3A aNcat GAPDH.jpg]

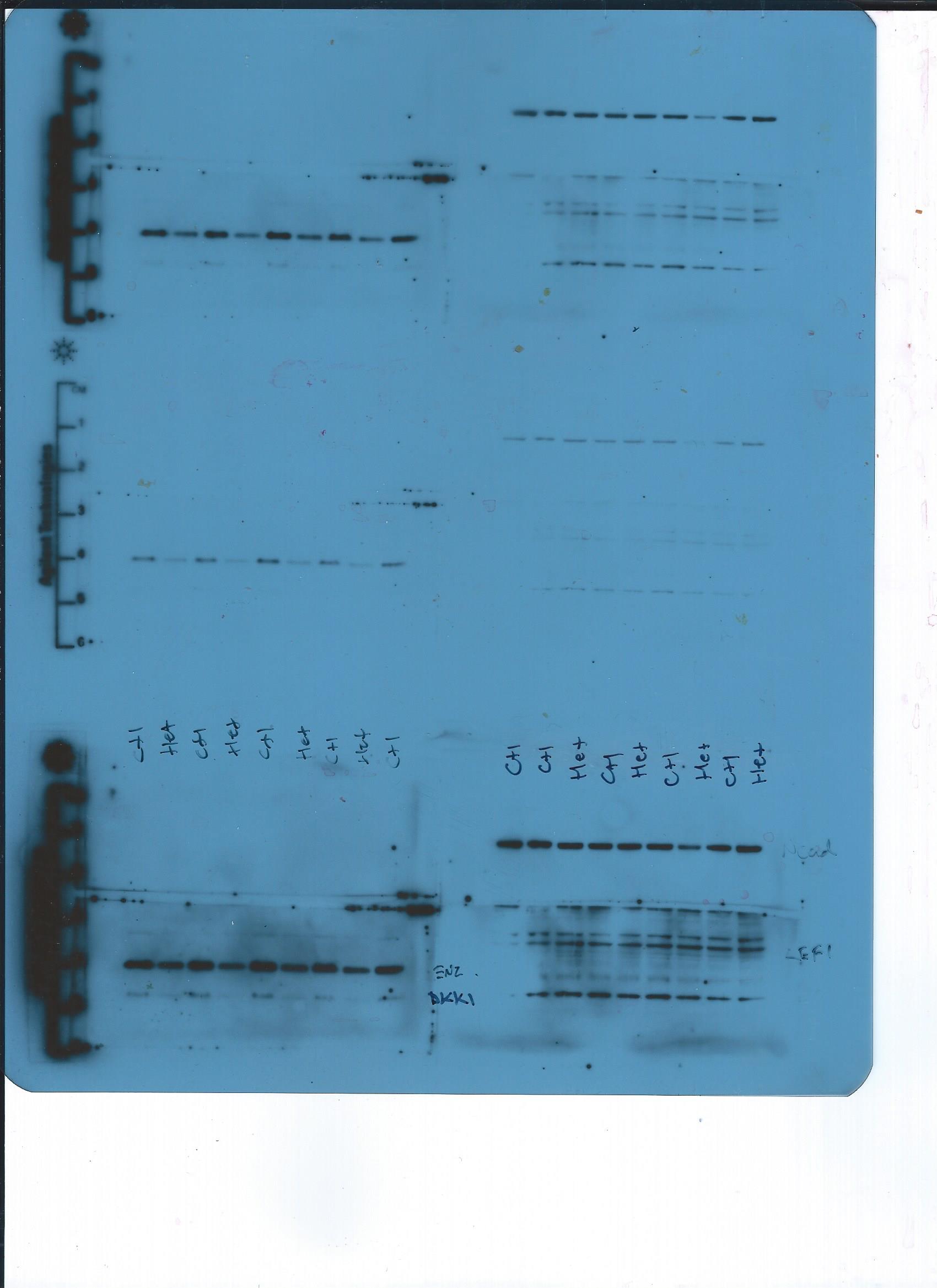

Supplement: Supplementary file 4 — Source data Fig. 3 [file 44321_2024_110_MOESM4_ESM.zip › 3A/3A-C Ncad LEF1 EN2 DKK1.jpg]

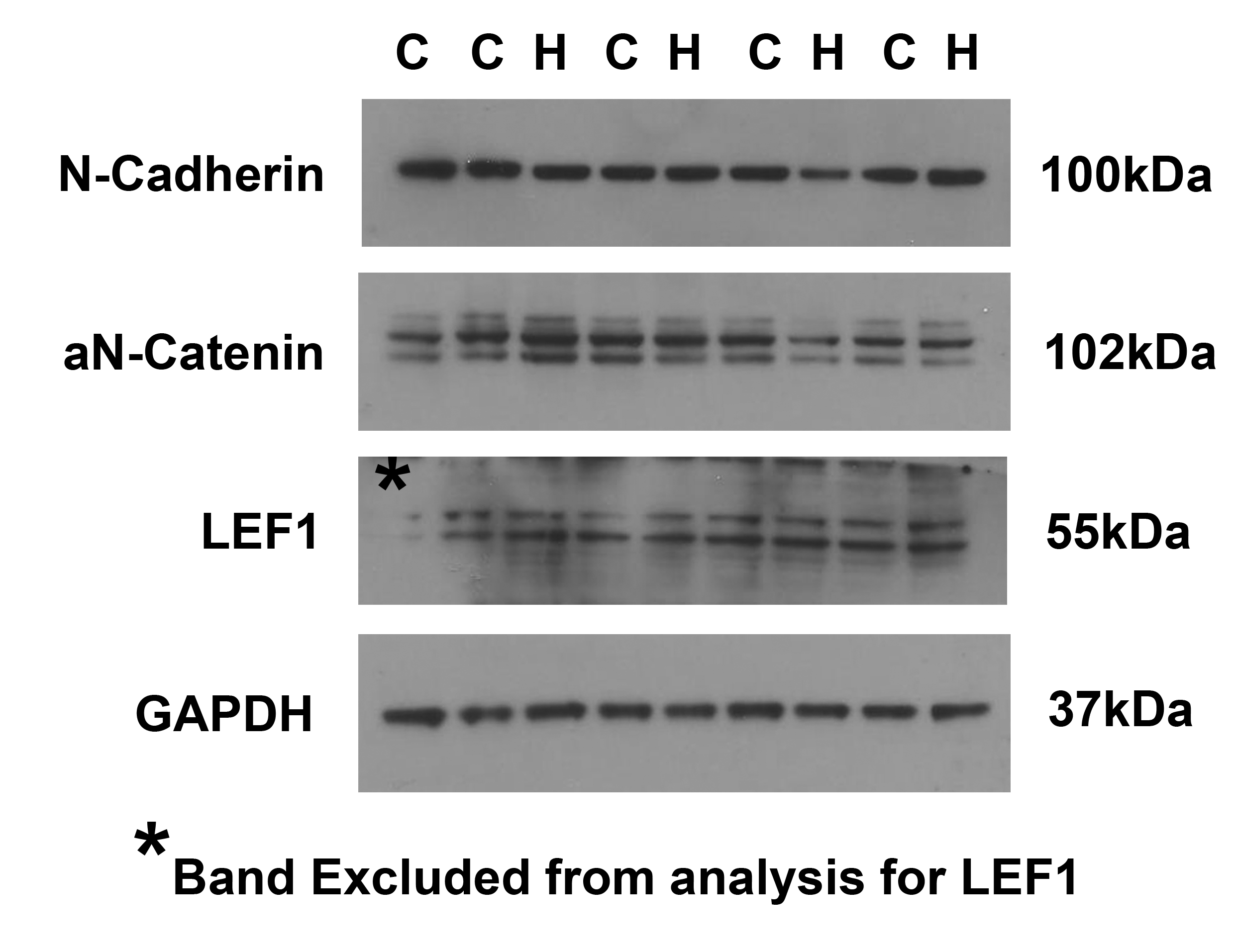

Supplement: Supplementary file 4 — Source data Fig. 3 [file 44321_2024_110_MOESM4_ESM.zip › 3A/Figure 3a Annotated.tif]

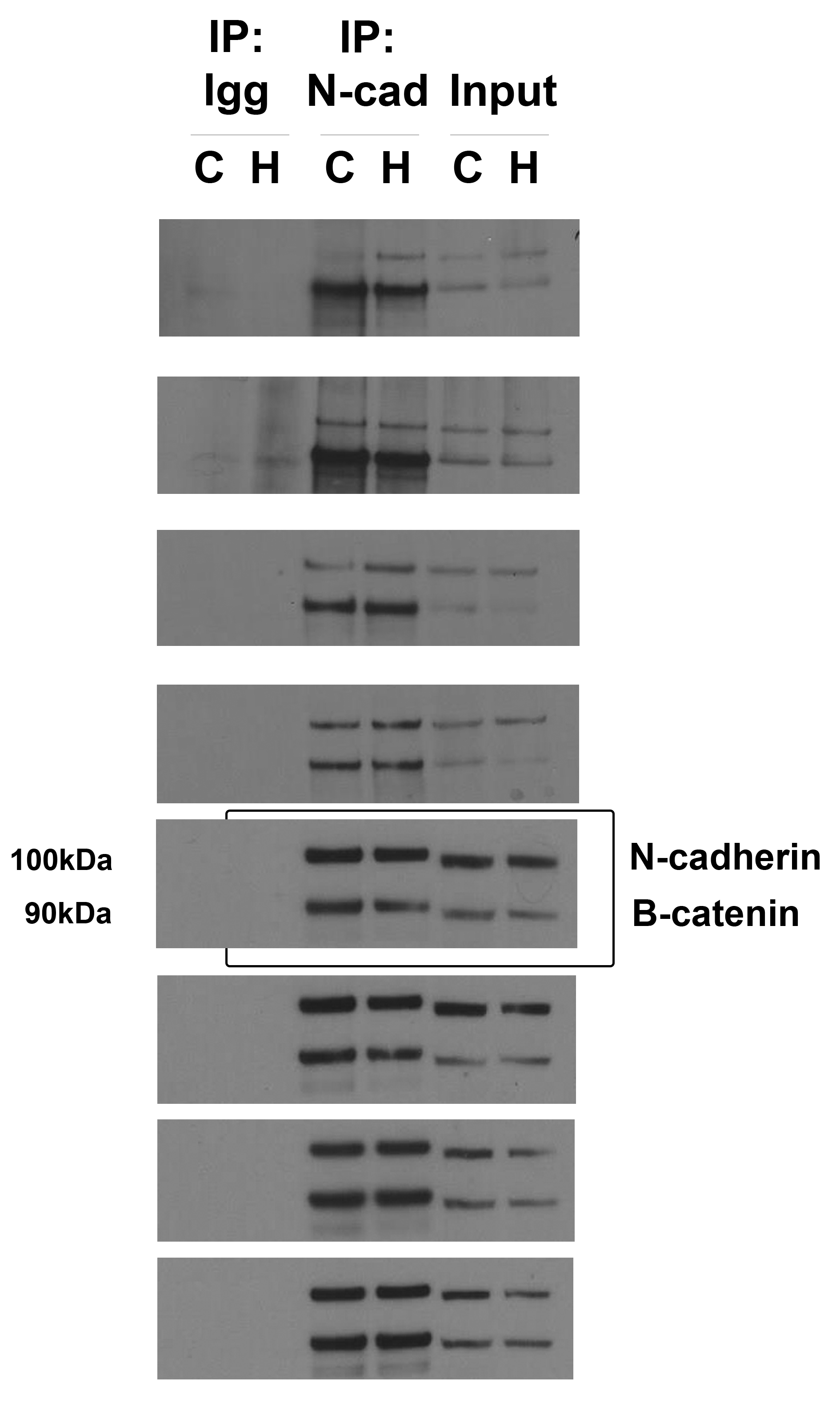

Supplement: Supplementary file 4 — Source data Fig. 3 [file 44321_2024_110_MOESM4_ESM.zip › 3B/Figure 3b Annotated.tif]

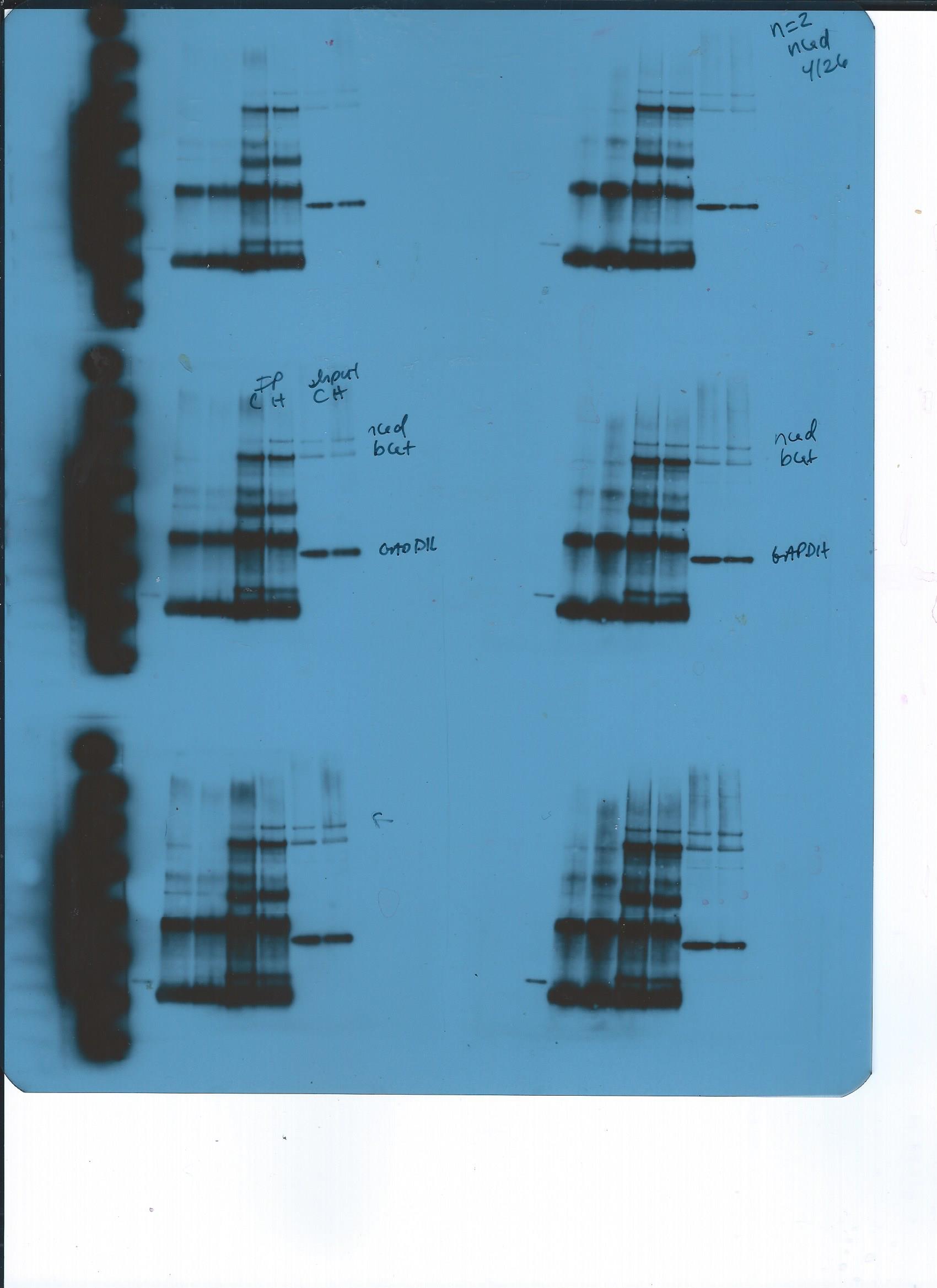

Supplement: Supplementary file 4 — Source data Fig. 3 [file 44321_2024_110_MOESM4_ESM.zip › 3B/N-cad IP Set 1-2.jpg]

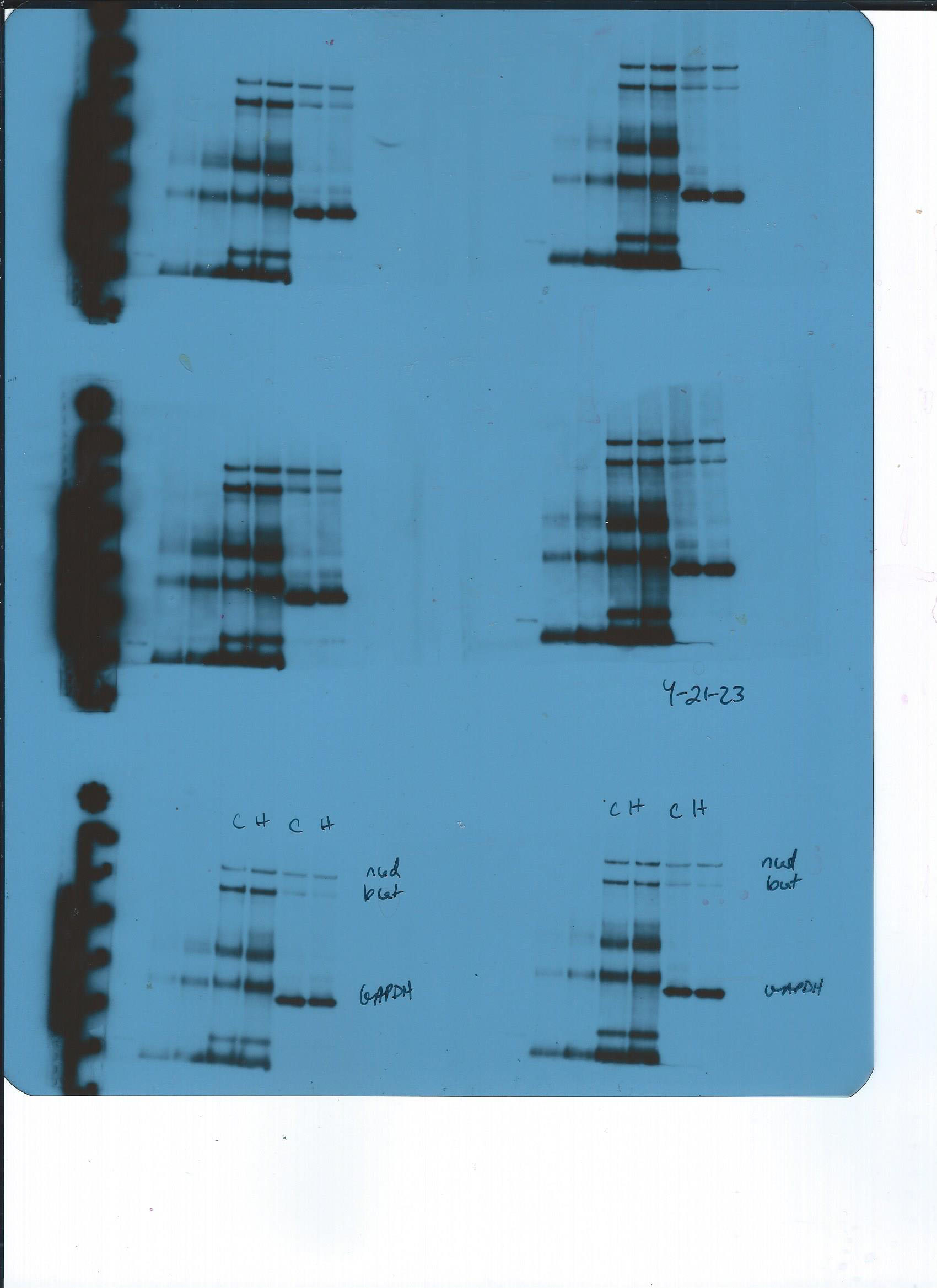

Supplement: Supplementary file 4 — Source data Fig. 3 [file 44321_2024_110_MOESM4_ESM.zip › 3B/N-cad IP Set 3-4.jpg]

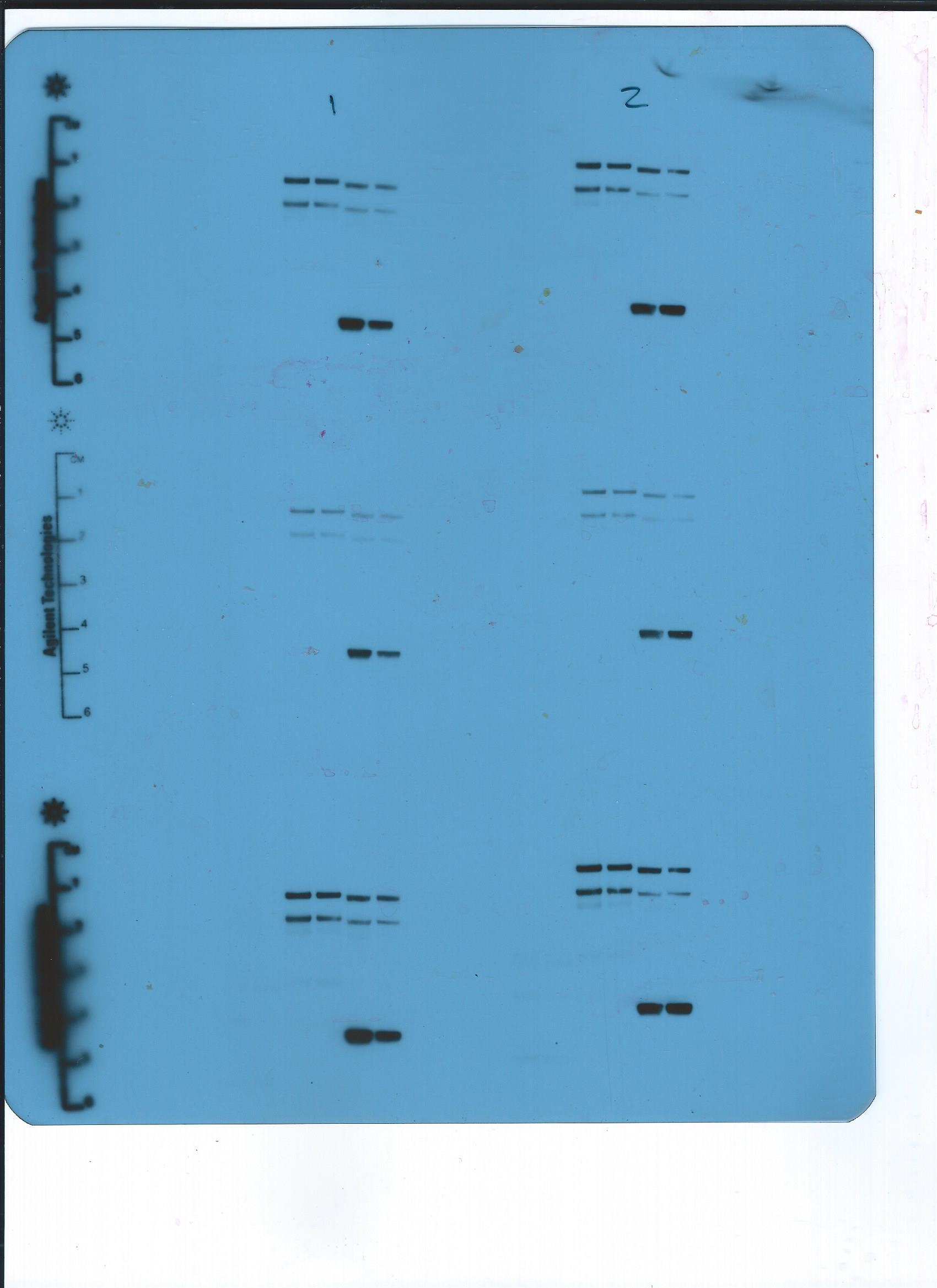

Supplement: Supplementary file 4 — Source data Fig. 3 [file 44321_2024_110_MOESM4_ESM.zip › 3B/N-cad IP Set 5-6.jpg]

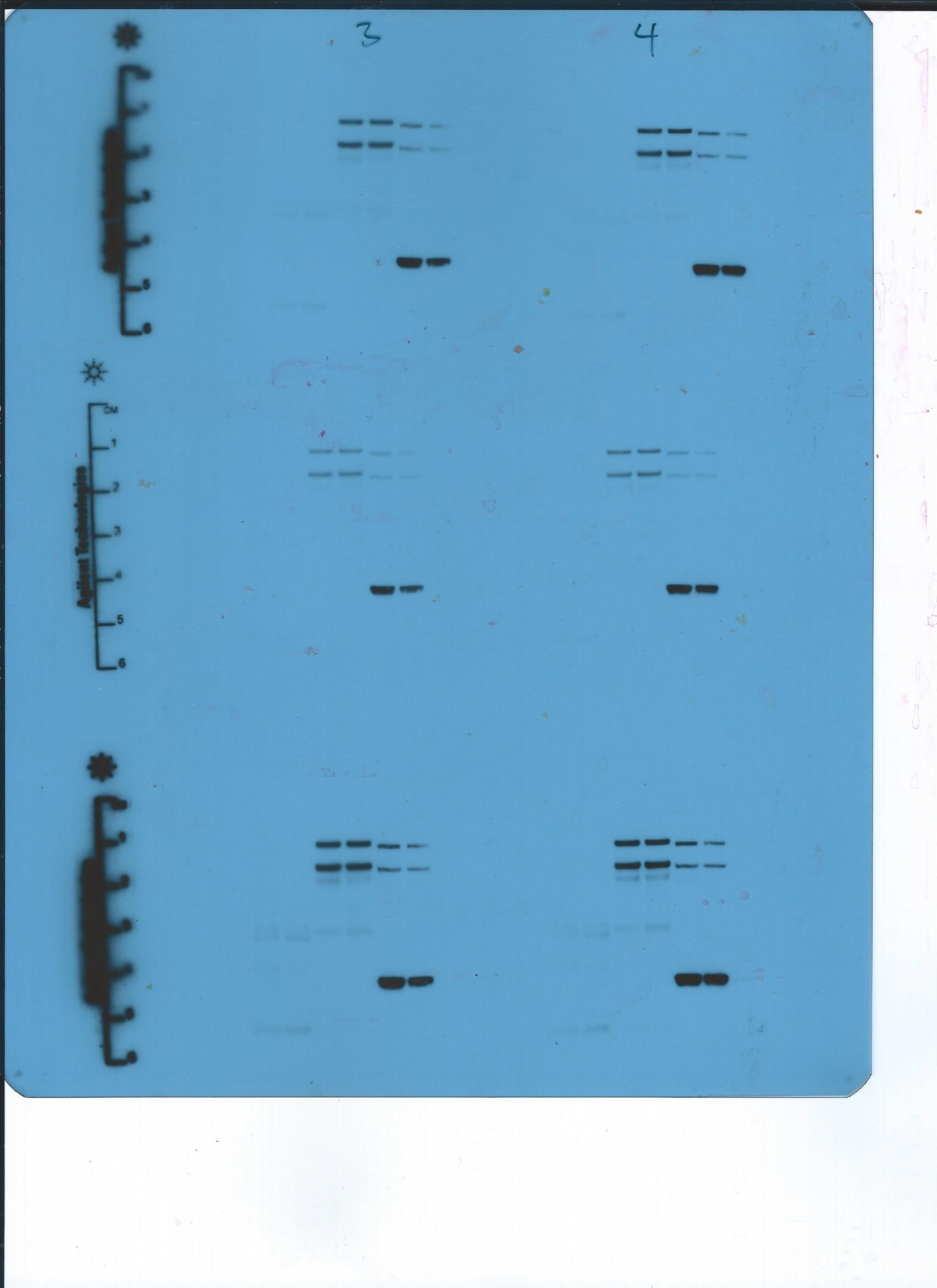

Supplement: Supplementary file 4 — Source data Fig. 3 [file 44321_2024_110_MOESM4_ESM.zip › 3B/N-cad IP Set 7-8.jpg]

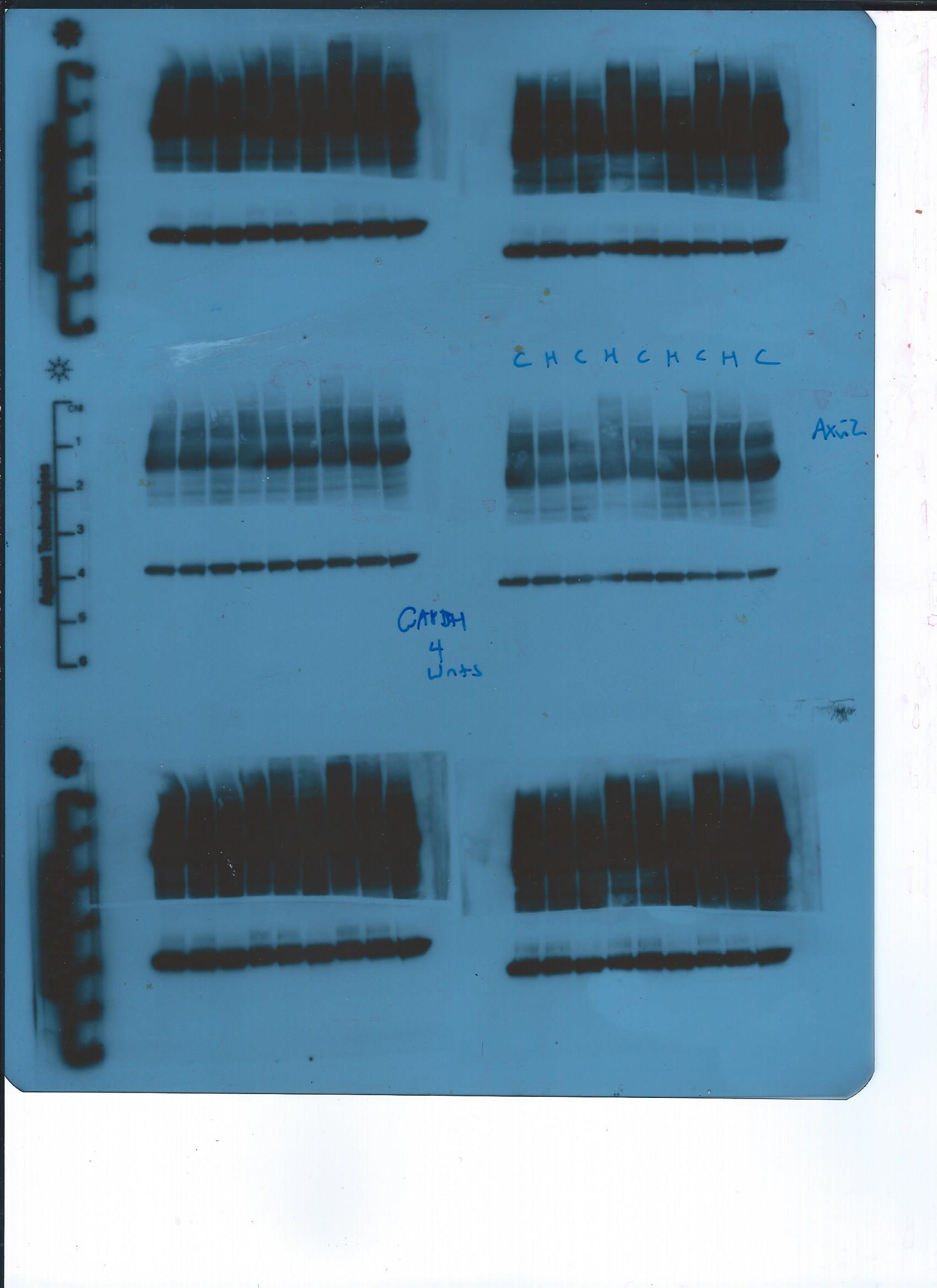

Supplement: Supplementary file 4 — Source data Fig. 3 [file 44321_2024_110_MOESM4_ESM.zip › 3C/3C GAPDH.jpg]

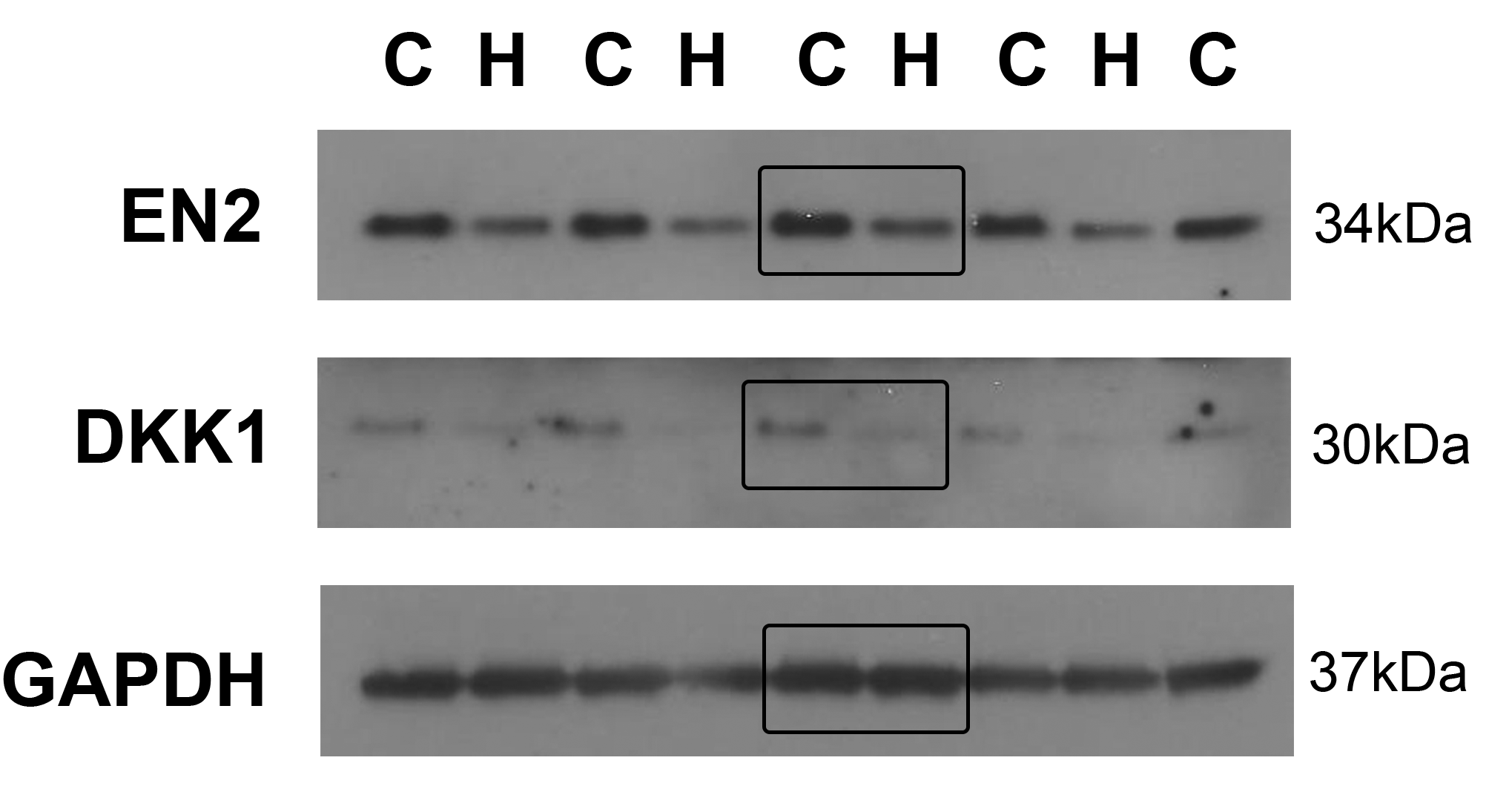

Supplement: Supplementary file 4 — Source data Fig. 3 [file 44321_2024_110_MOESM4_ESM.zip › 3C/Figure 3c Annotated.tif]

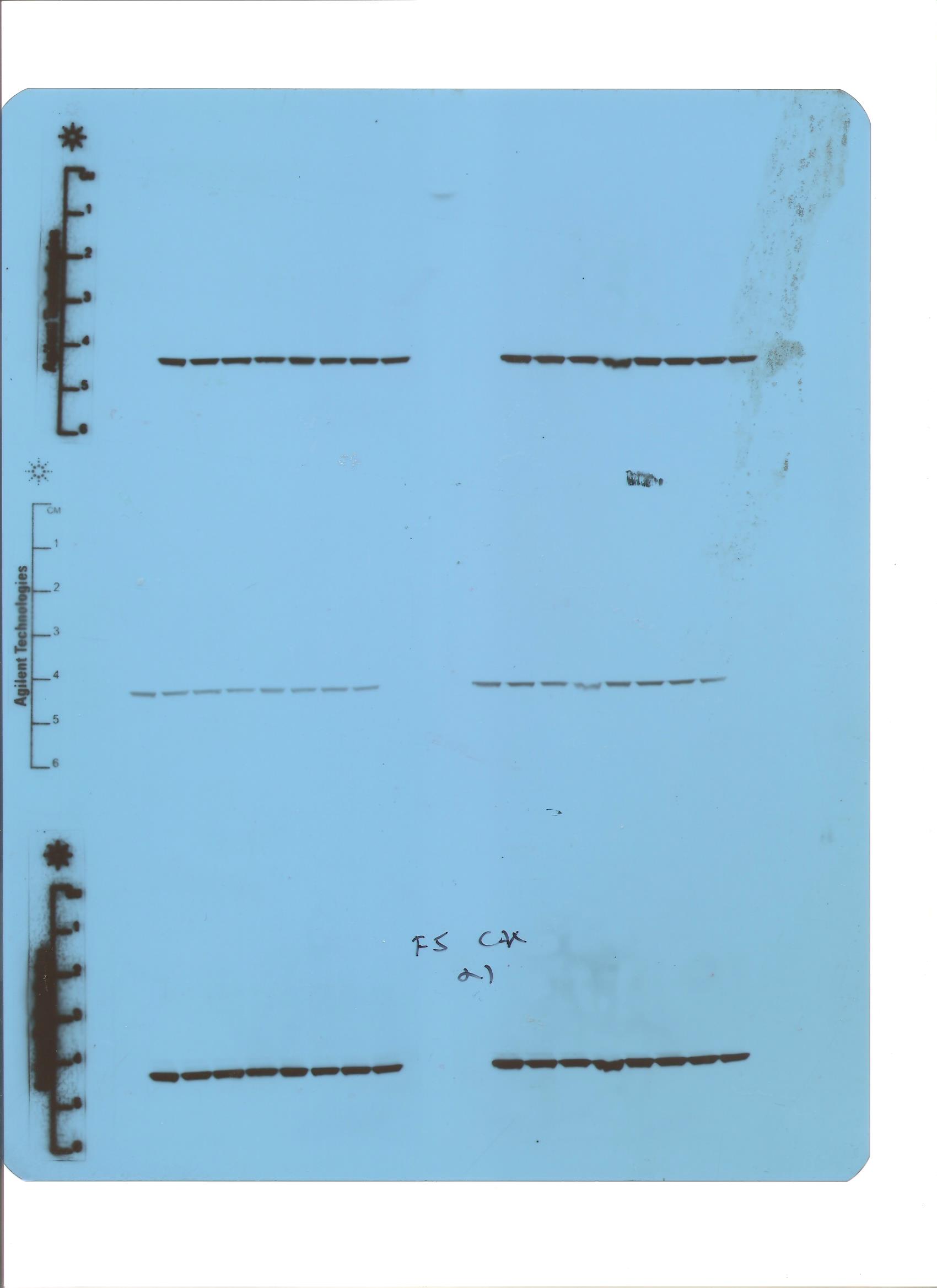

Supplement: Supplementary file 6 — Source data Fig. 5 [file 44321_2024_110_MOESM6_ESM.zip › Fig 5/F5 Ctx a1.jpg]

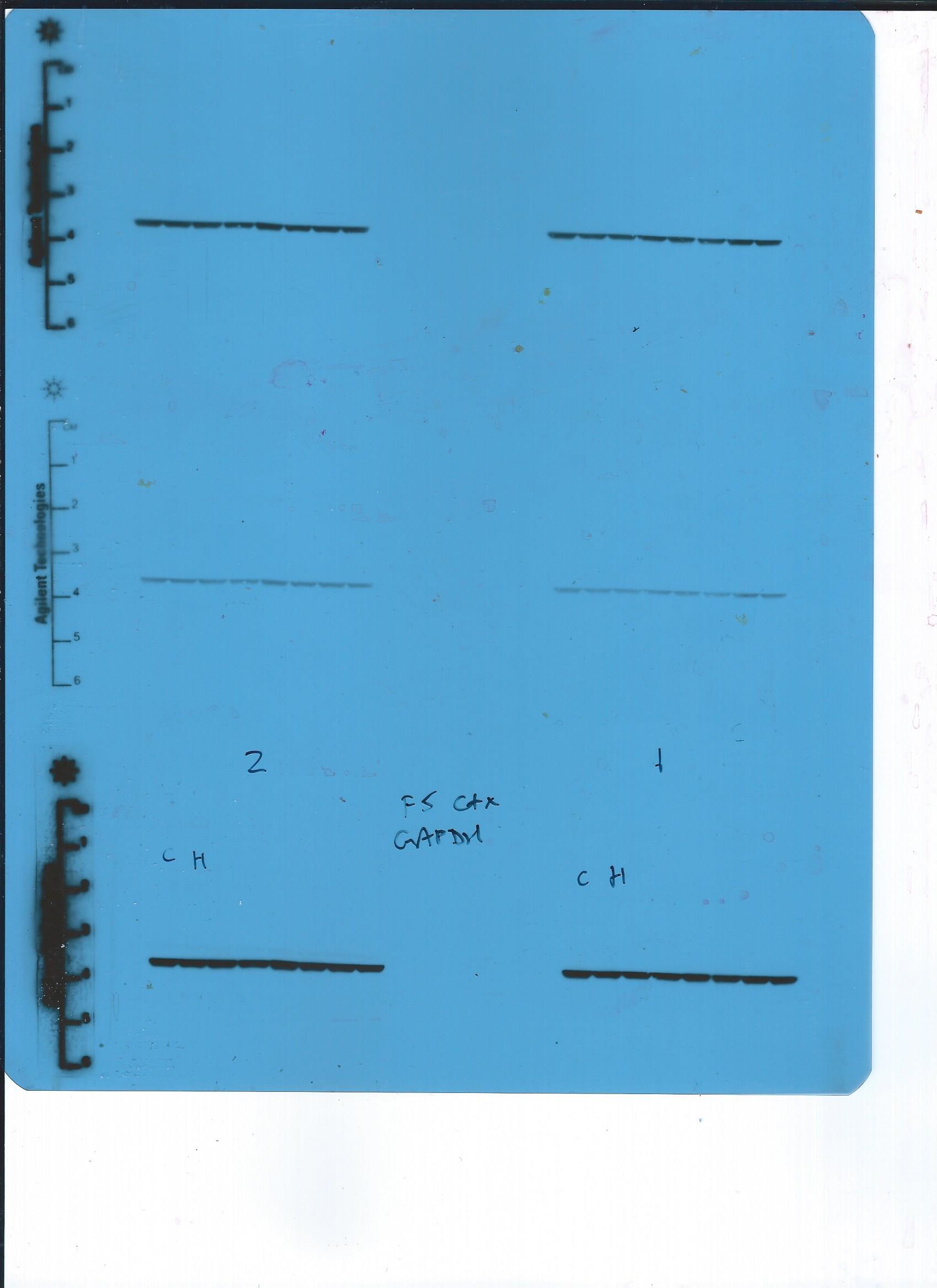

Supplement: Supplementary file 6 — Source data Fig. 5 [file 44321_2024_110_MOESM6_ESM.zip › Fig 5/F5 Ctx GAPDH.jpg]

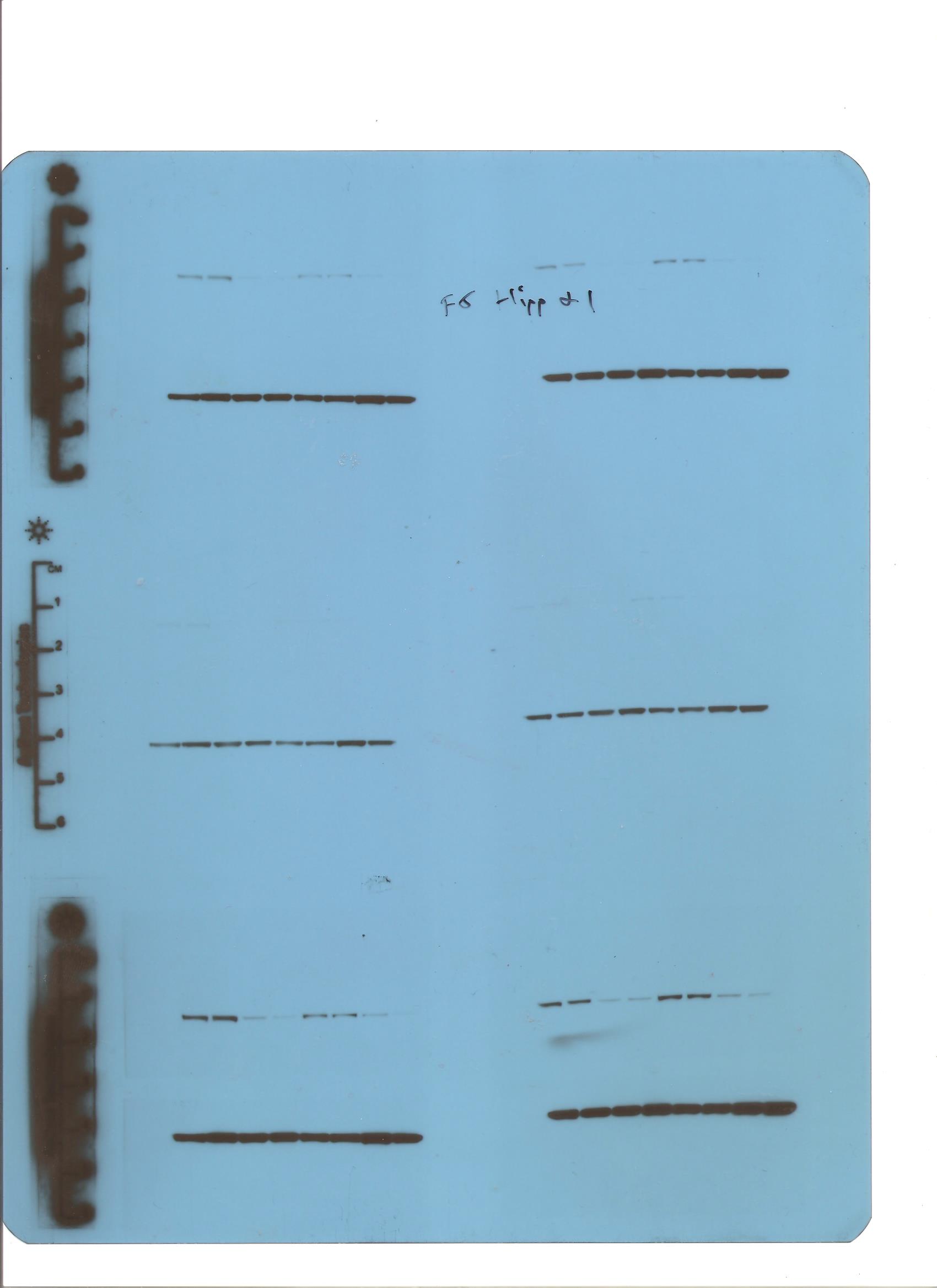

Supplement: Supplementary file 6 — Source data Fig. 5 [file 44321_2024_110_MOESM6_ESM.zip › Fig 5/F5 Hipp a1.jpg]

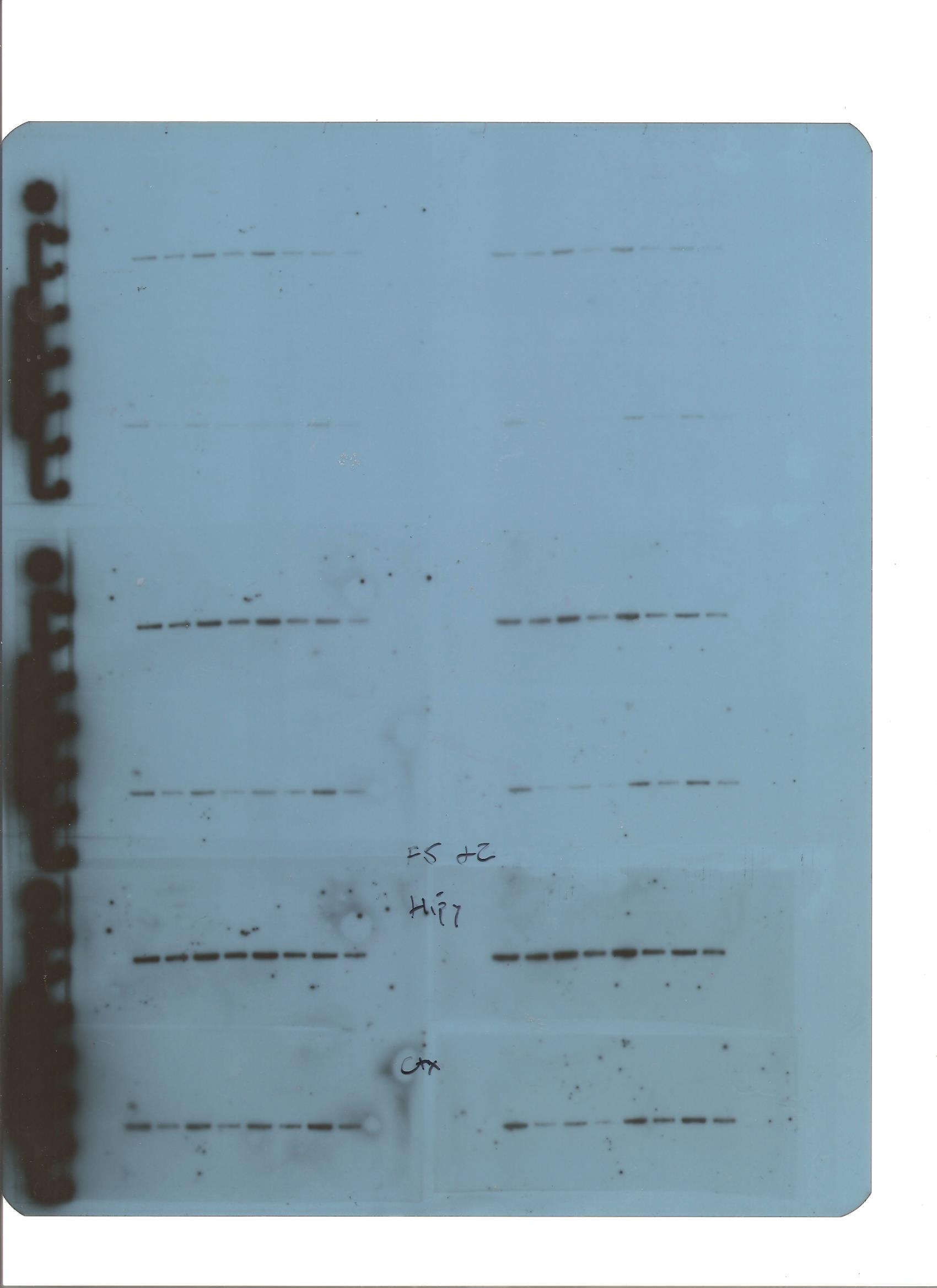

Supplement: Supplementary file 6 — Source data Fig. 5 [file 44321_2024_110_MOESM6_ESM.zip › Fig 5/F5 Hipp Ctx a2.jpg]

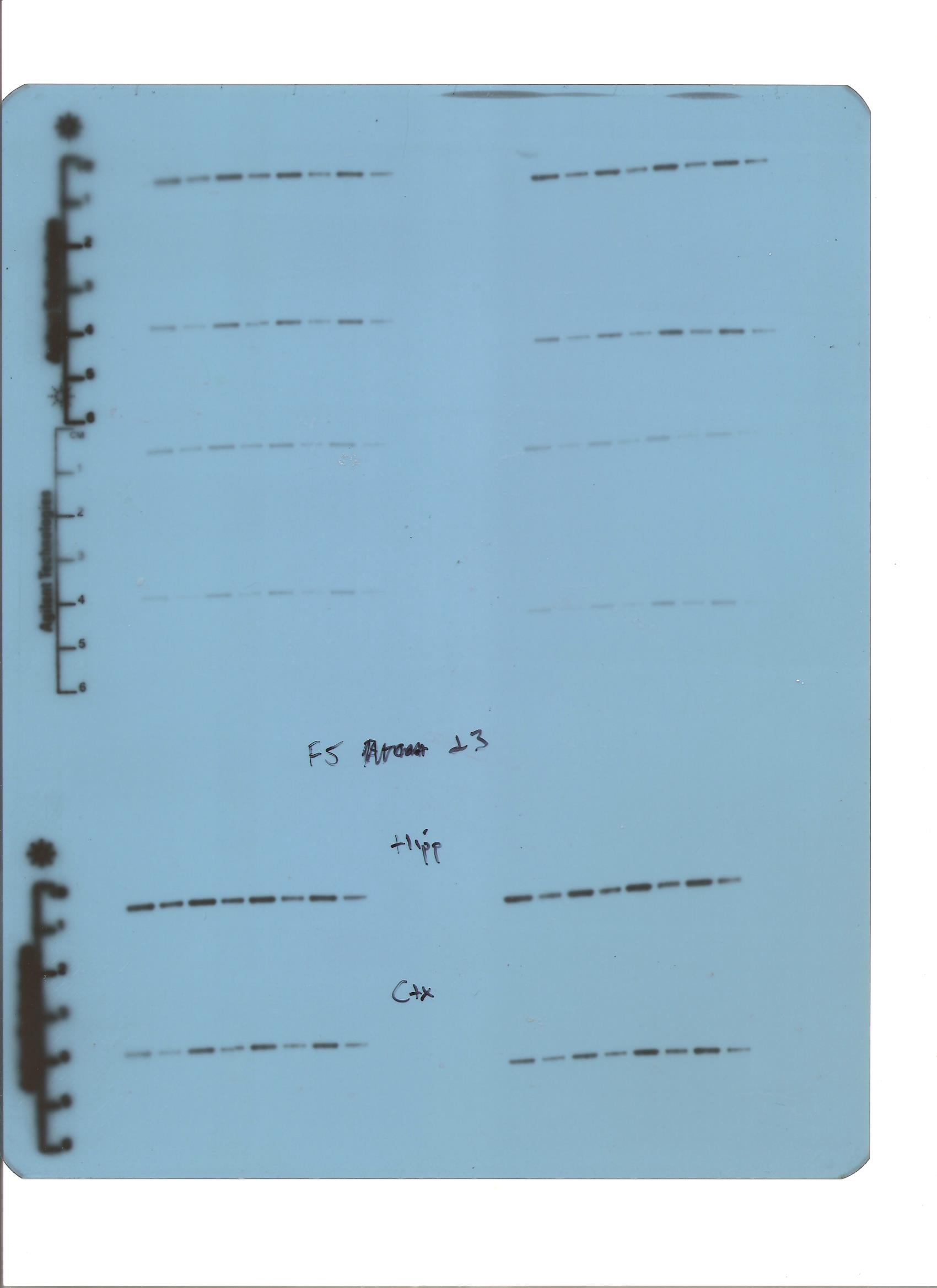

Supplement: Supplementary file 6 — Source data Fig. 5 [file 44321_2024_110_MOESM6_ESM.zip › Fig 5/F5 Hipp Ctx a3.jpg]

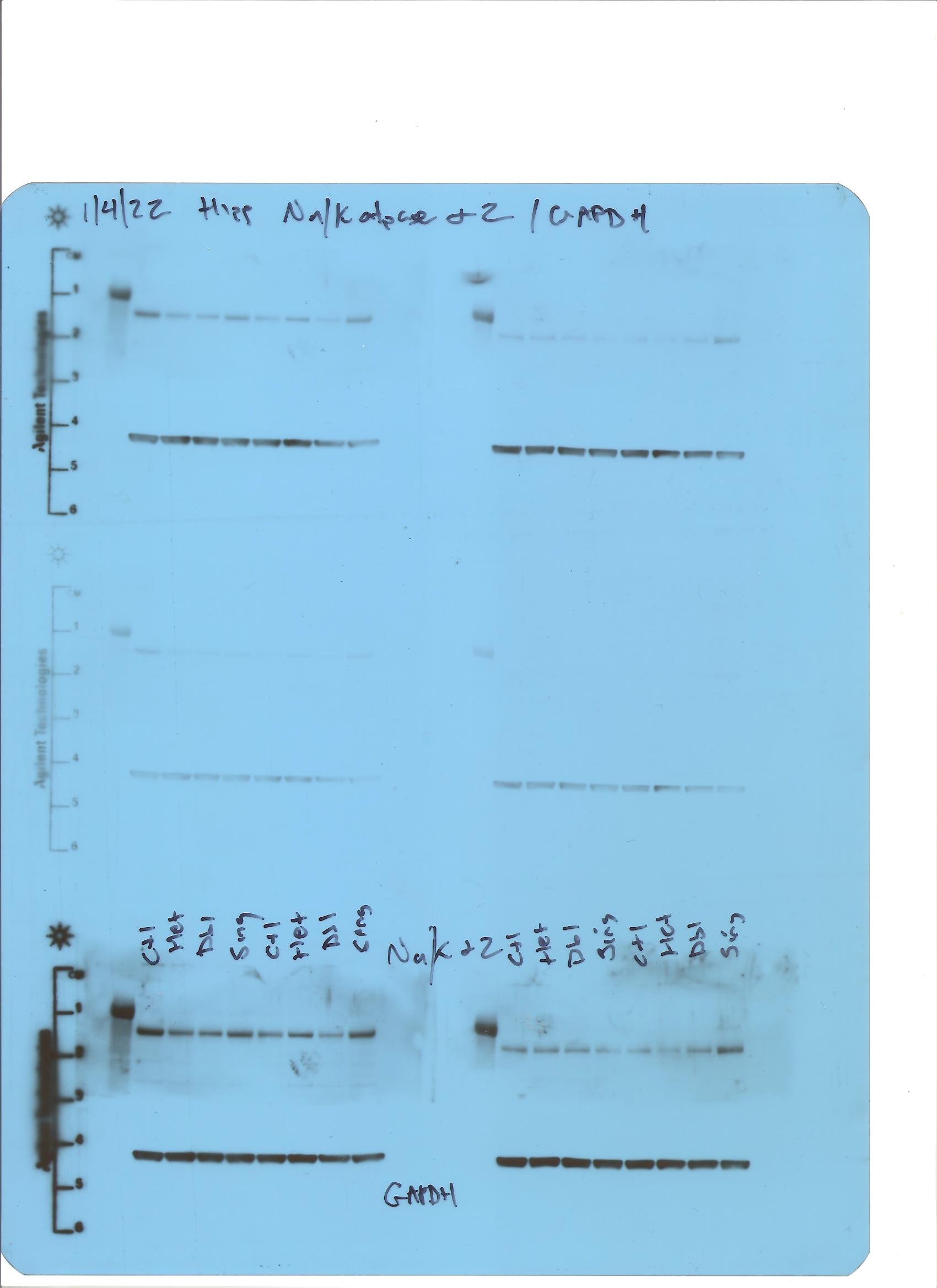

Supplement: Supplementary file 6 — Source data Fig. 5 [file 44321_2024_110_MOESM6_ESM.zip › Fig 5/F5 Hipp GAPDH.jpeg]

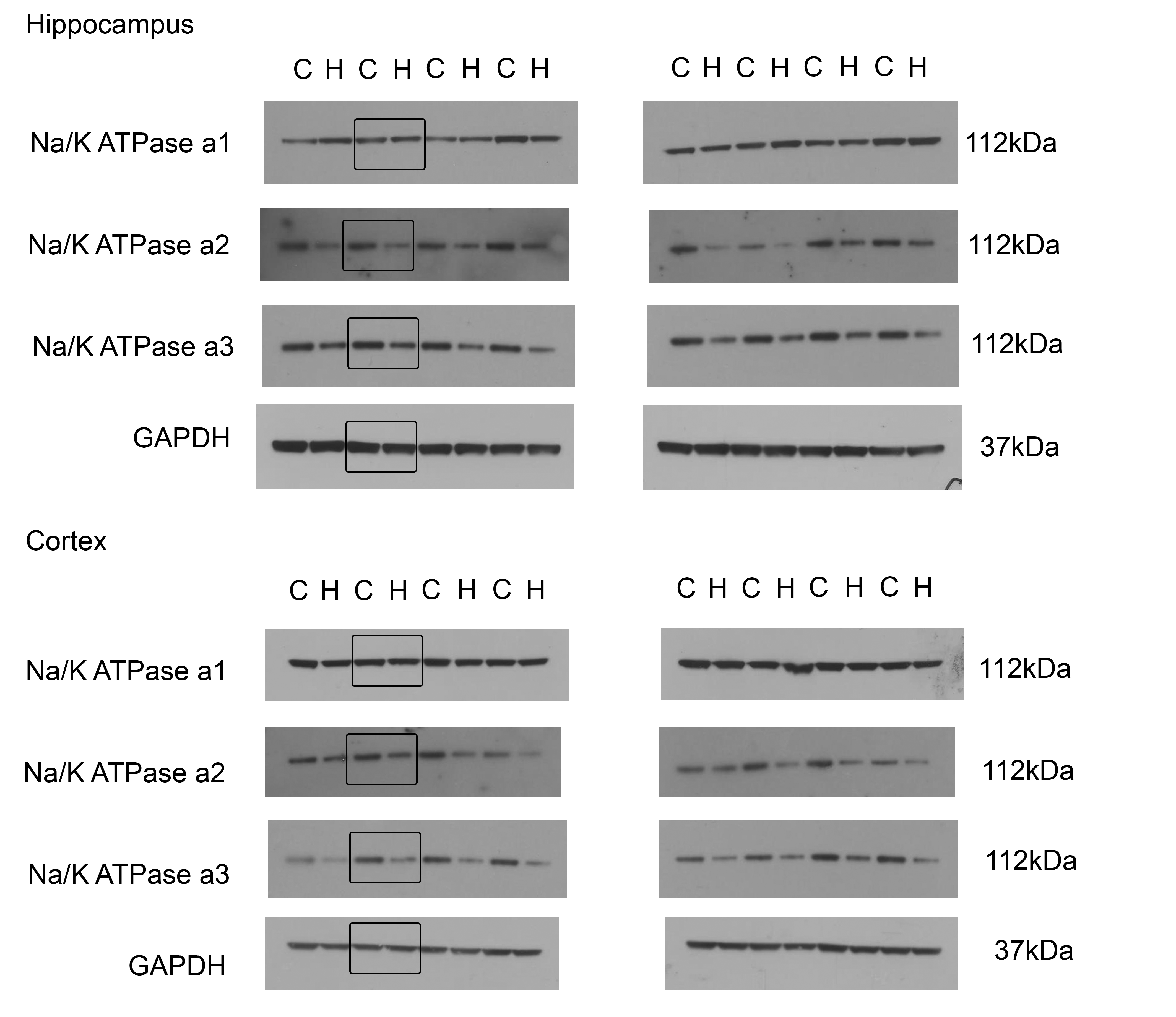

Supplement: Supplementary file 6 — Source data Fig. 5 [file 44321_2024_110_MOESM6_ESM.zip › Fig 5/Figure 5 Westerns Annotated.tif]

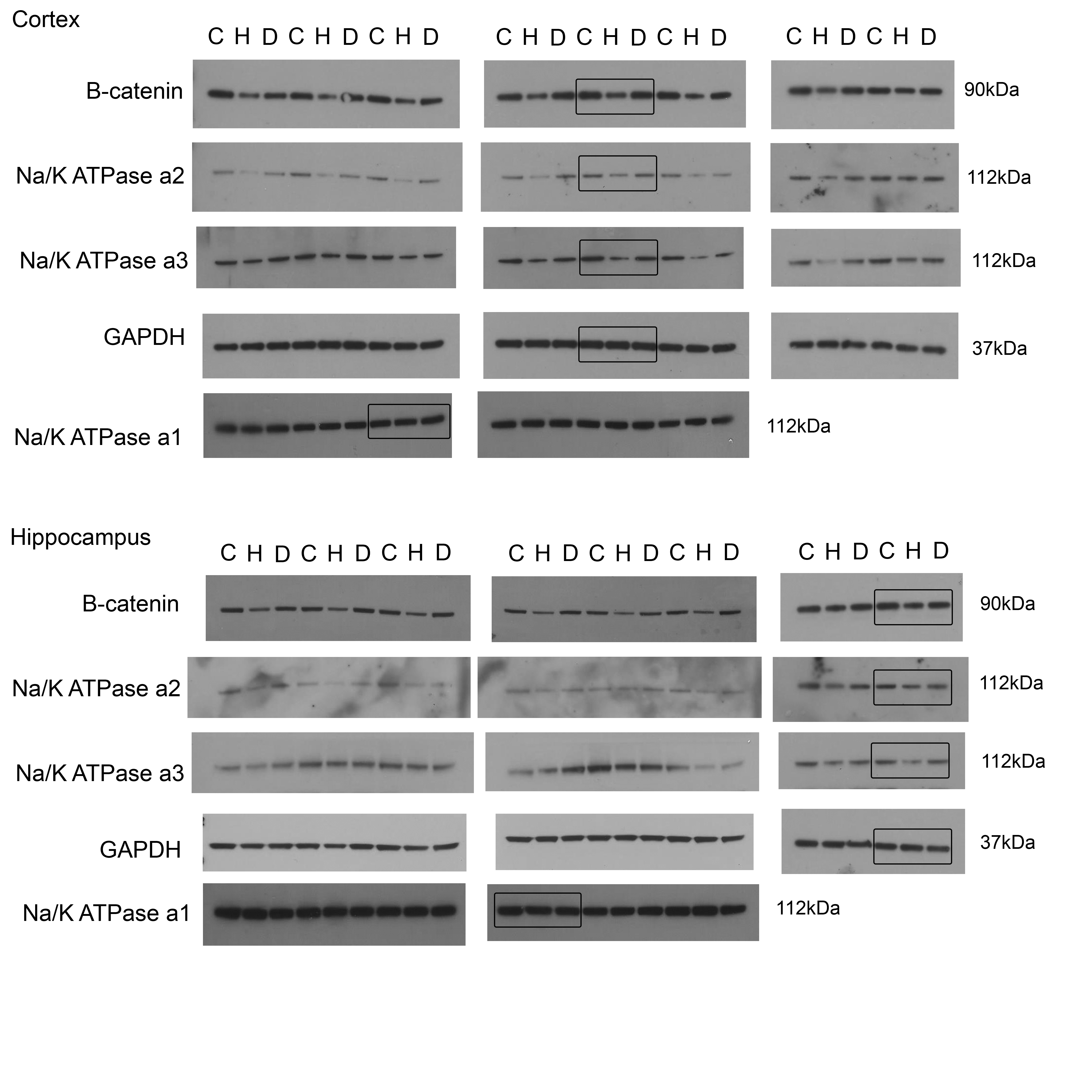

Supplement: Supplementary file 7 — Source data Fig. 6 [file 44321_2024_110_MOESM7_ESM.zip › 6B/6A Annotated Western.tif]

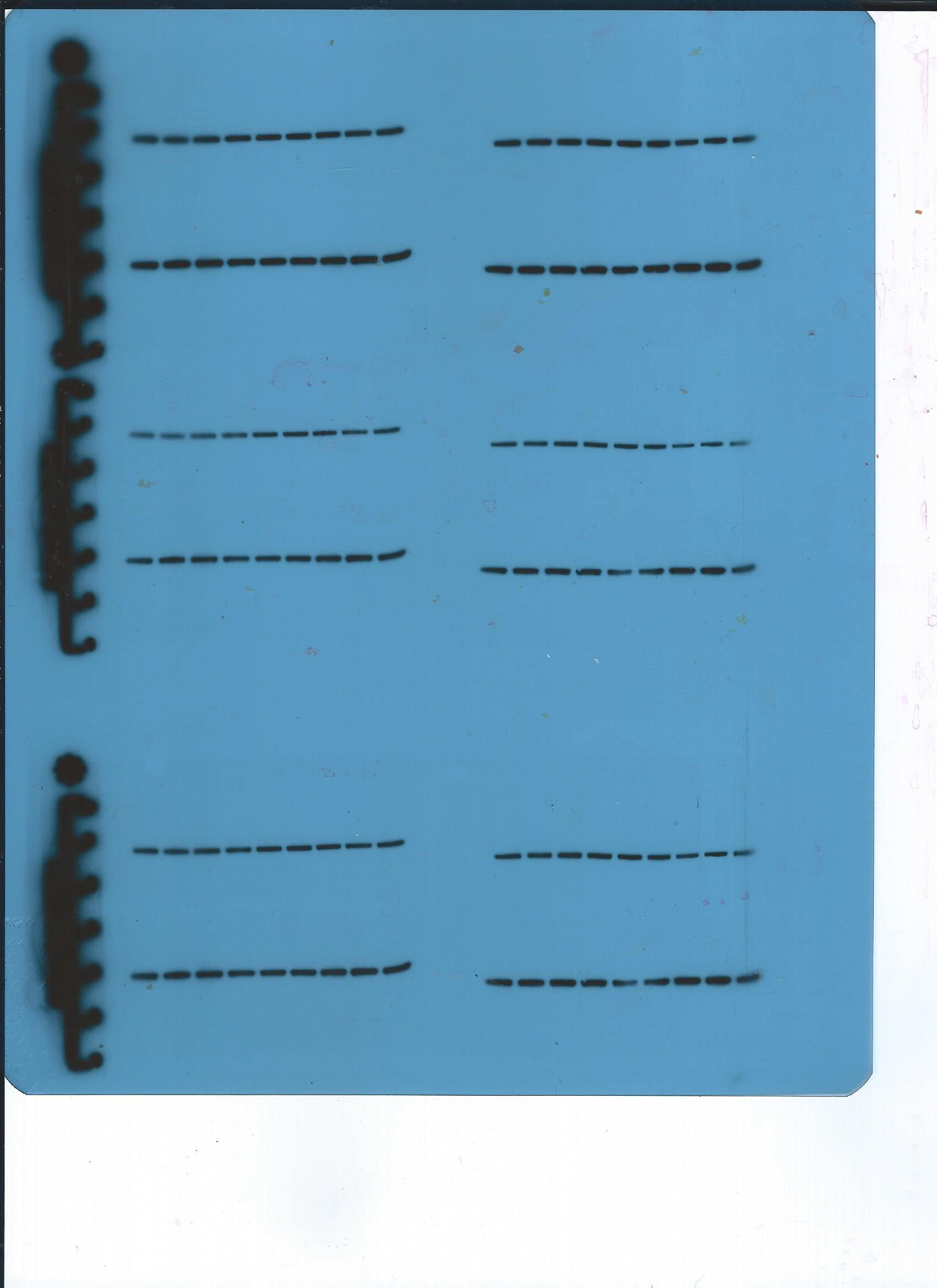

Supplement: Supplementary file 7 — Source data Fig. 6 [file 44321_2024_110_MOESM7_ESM.zip › 6B/6A ATP1A1 GAPDH BRD0320 Ctx.jpg]

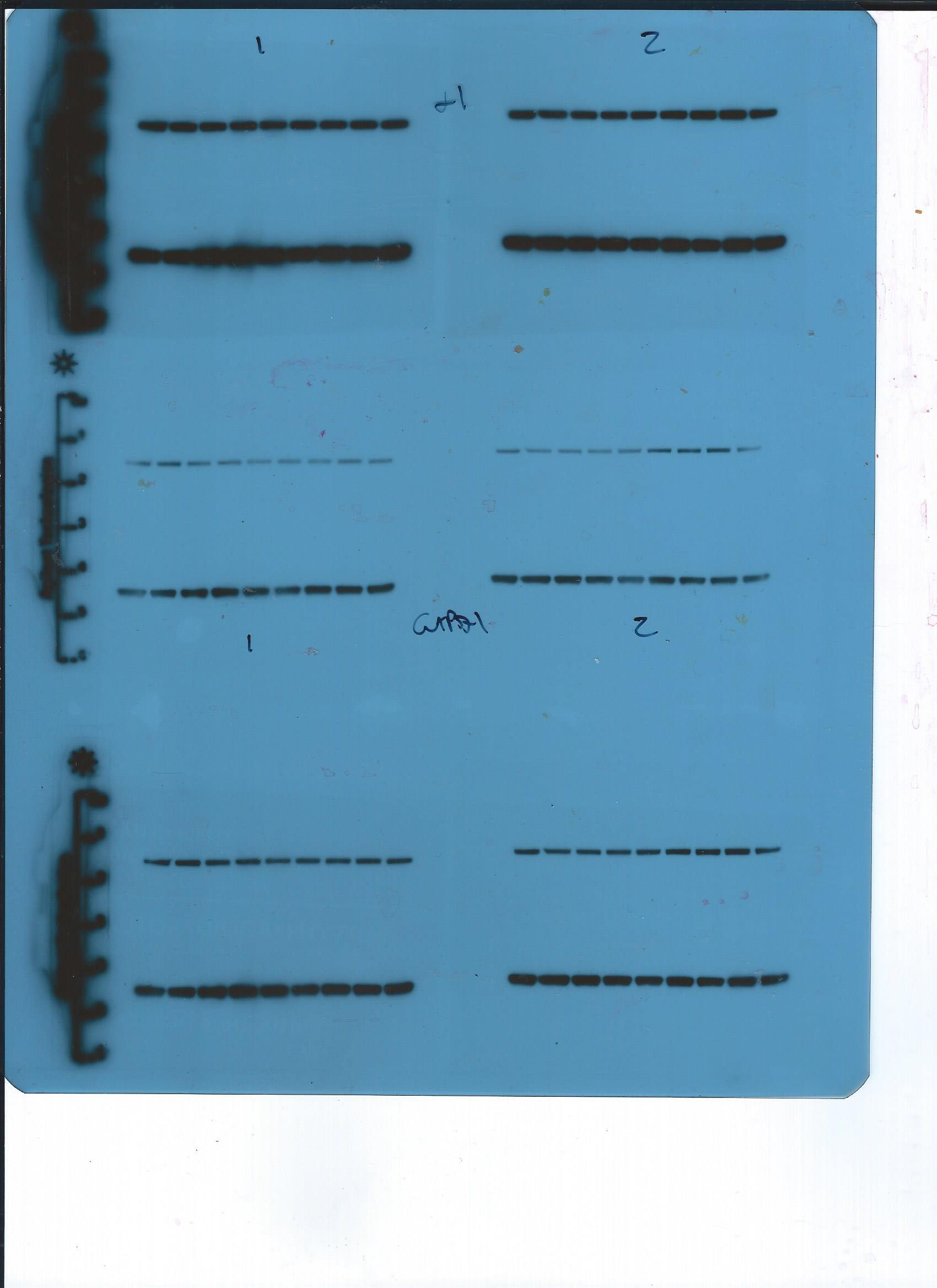

Supplement: Supplementary file 7 — Source data Fig. 6 [file 44321_2024_110_MOESM7_ESM.zip › 6B/6A ATP1A1 GAPDH BRD0320 Hip.jpg]

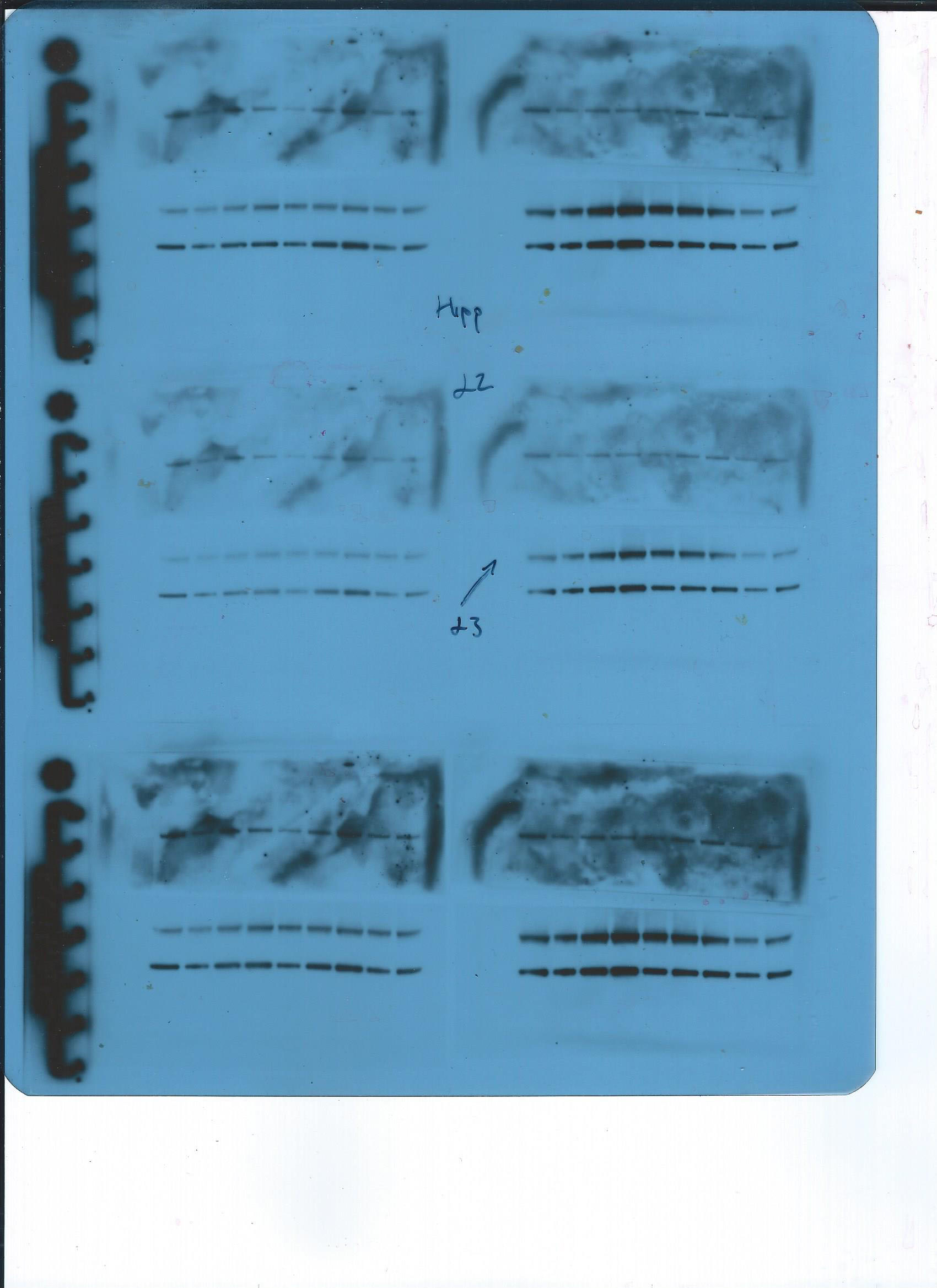

Supplement: Supplementary file 7 — Source data Fig. 6 [file 44321_2024_110_MOESM7_ESM.zip › 6B/6A ATP1A2 ATP1A3 BRD0320 Hipp.jpg]

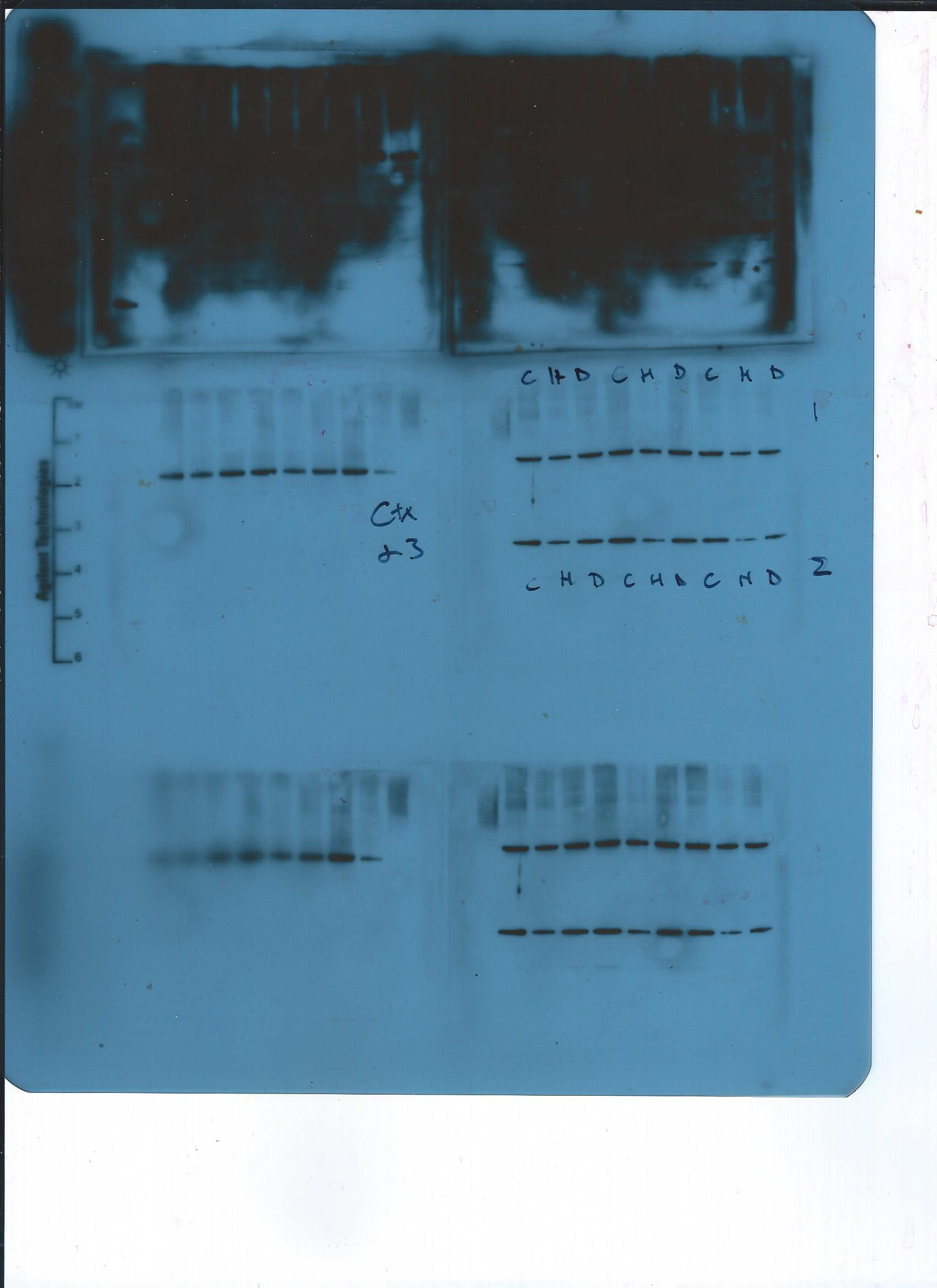

Supplement: Supplementary file 7 — Source data Fig. 6 [file 44321_2024_110_MOESM7_ESM.zip › 6B/6A ATP1A3 BRD0320 Ctx.jpg]

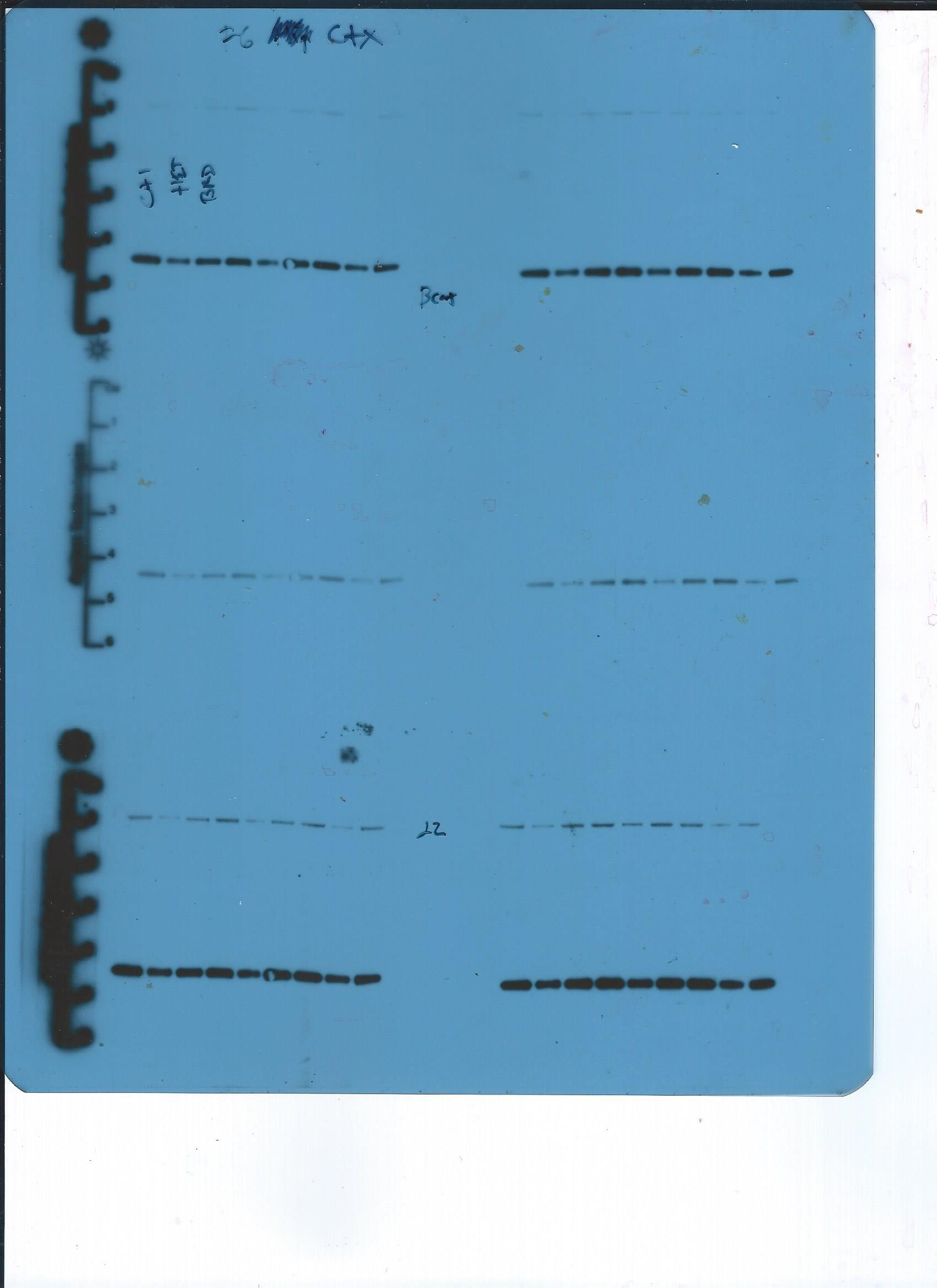

Supplement: Supplementary file 7 — Source data Fig. 6 [file 44321_2024_110_MOESM7_ESM.zip › 6B/6A Bcat ATP1A2 BRD0320 Ctx.jpg]

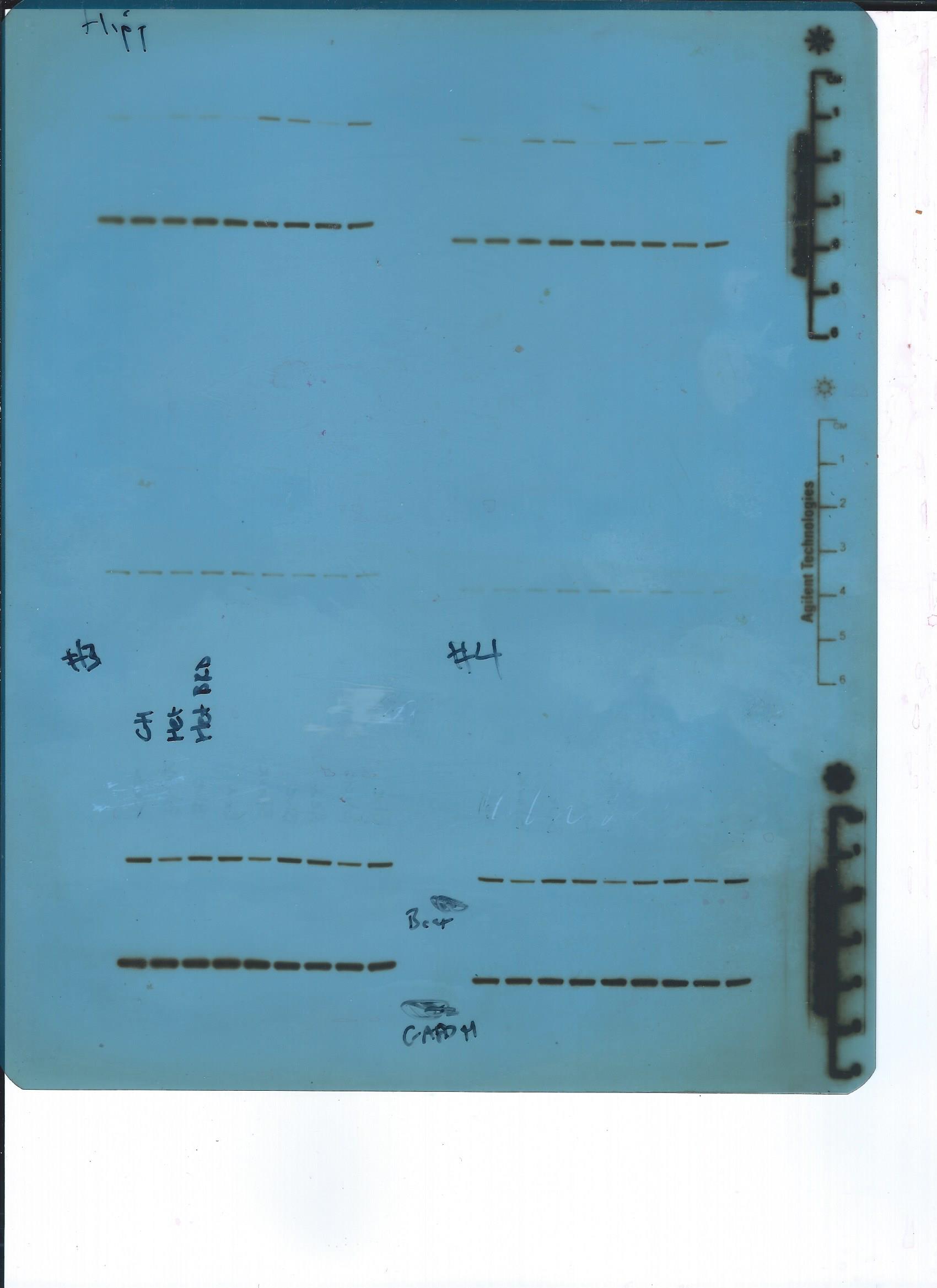

Supplement: Supplementary file 7 — Source data Fig. 6 [file 44321_2024_110_MOESM7_ESM.zip › 6B/6A Bcat BRD0320 Hipp.jpg]

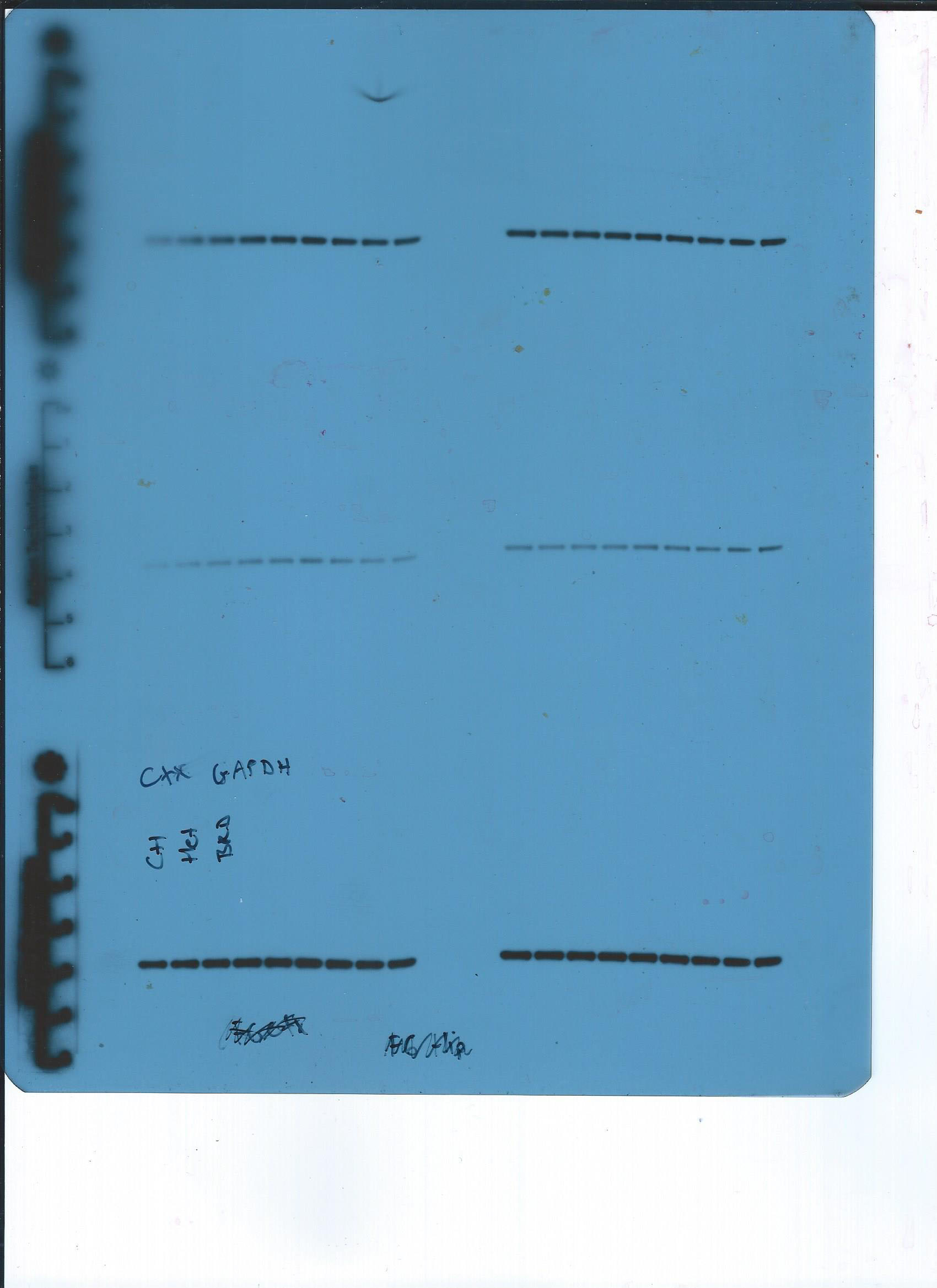

Supplement: Supplementary file 7 — Source data Fig. 6 [file 44321_2024_110_MOESM7_ESM.zip › 6B/6A GAPDH BRD0320 Ctx.jpg]

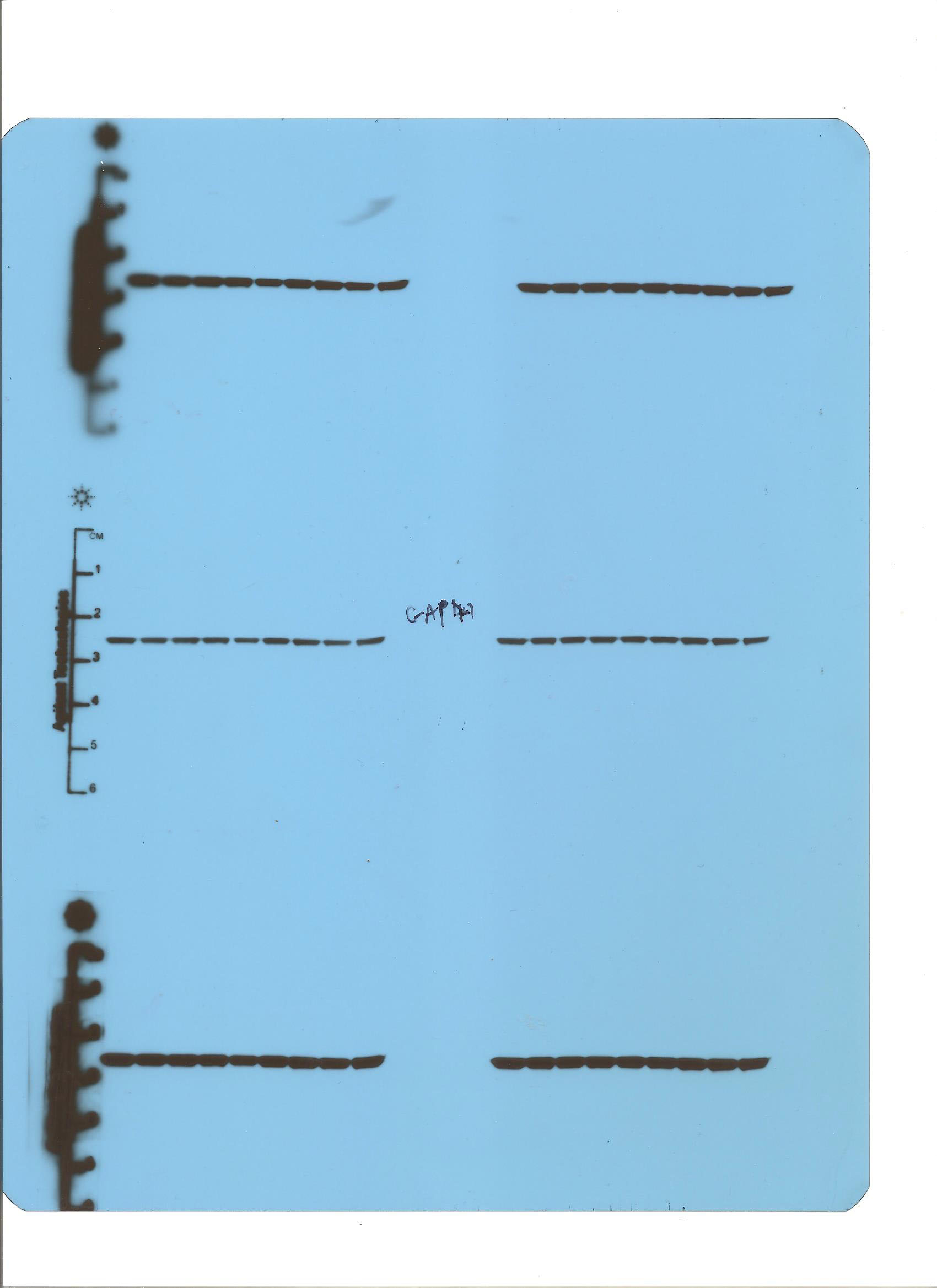

Supplement: Supplementary file 7 — Source data Fig. 6 [file 44321_2024_110_MOESM7_ESM.zip › 6B/6A GAPDH BRD0320 Hipp.jpg]

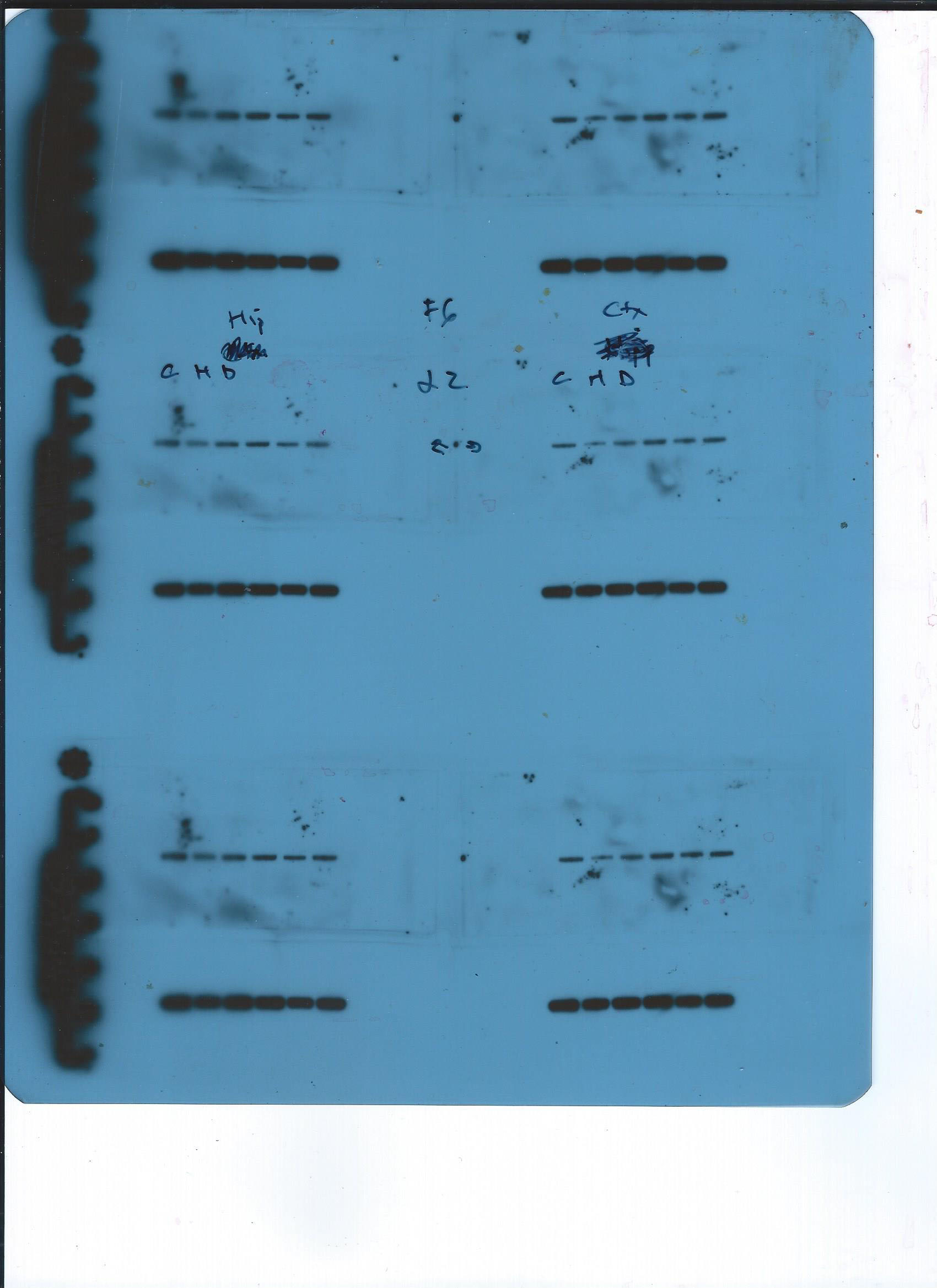

Supplement: Supplementary file 7 — Source data Fig. 6 [file 44321_2024_110_MOESM7_ESM.zip › 6B/6A n2 ctx and hipp ATP1A2 BRD0320.jpg]

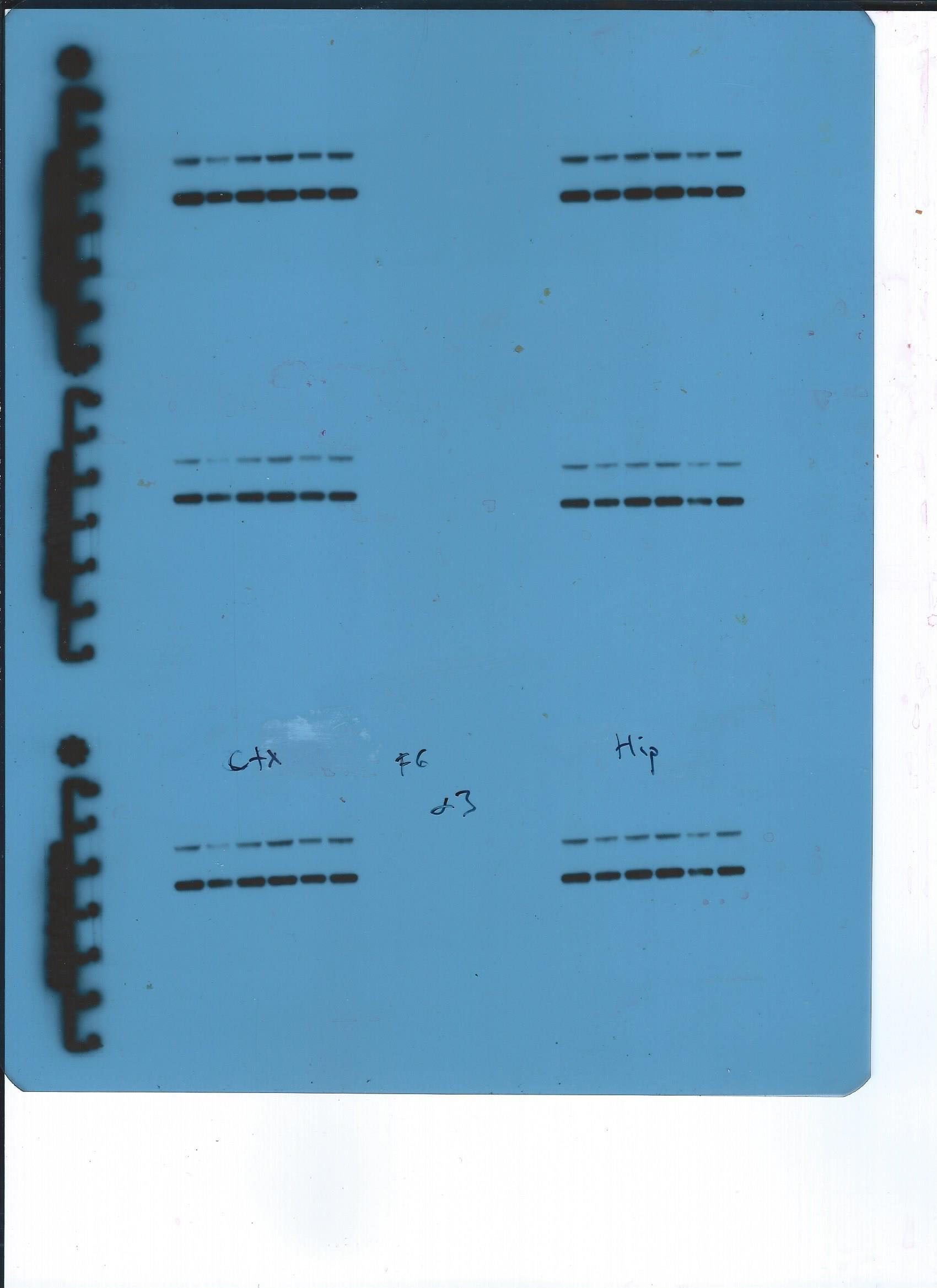

Supplement: Supplementary file 7 — Source data Fig. 6 [file 44321_2024_110_MOESM7_ESM.zip › 6B/6A n2 ctx and hipp ATP1A3 BRD0320.jpg]

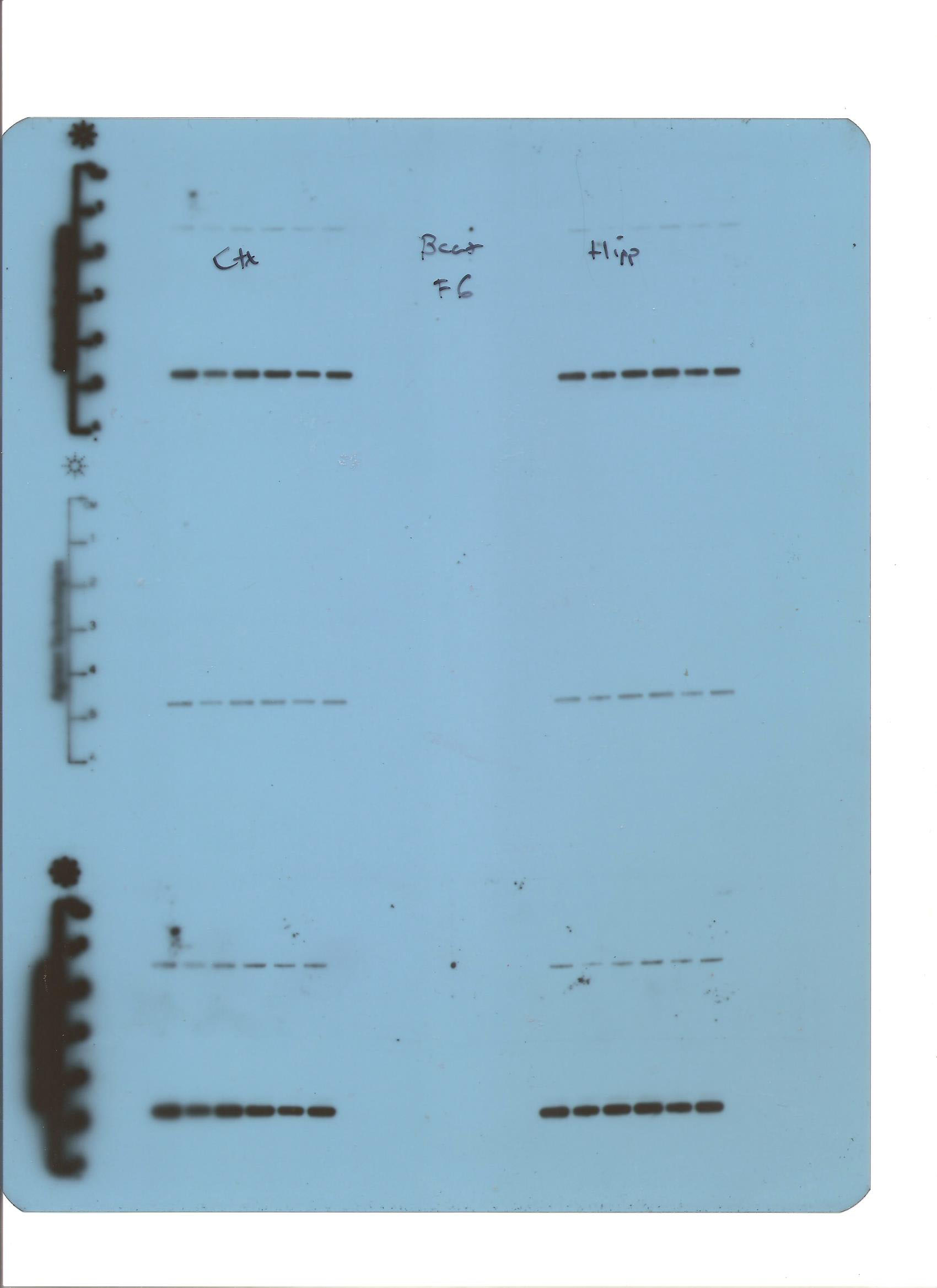

Supplement: Supplementary file 7 — Source data Fig. 6 [file 44321_2024_110_MOESM7_ESM.zip › 6B/6A n2 ctx and hipp Bcat BRD0320.jpg]

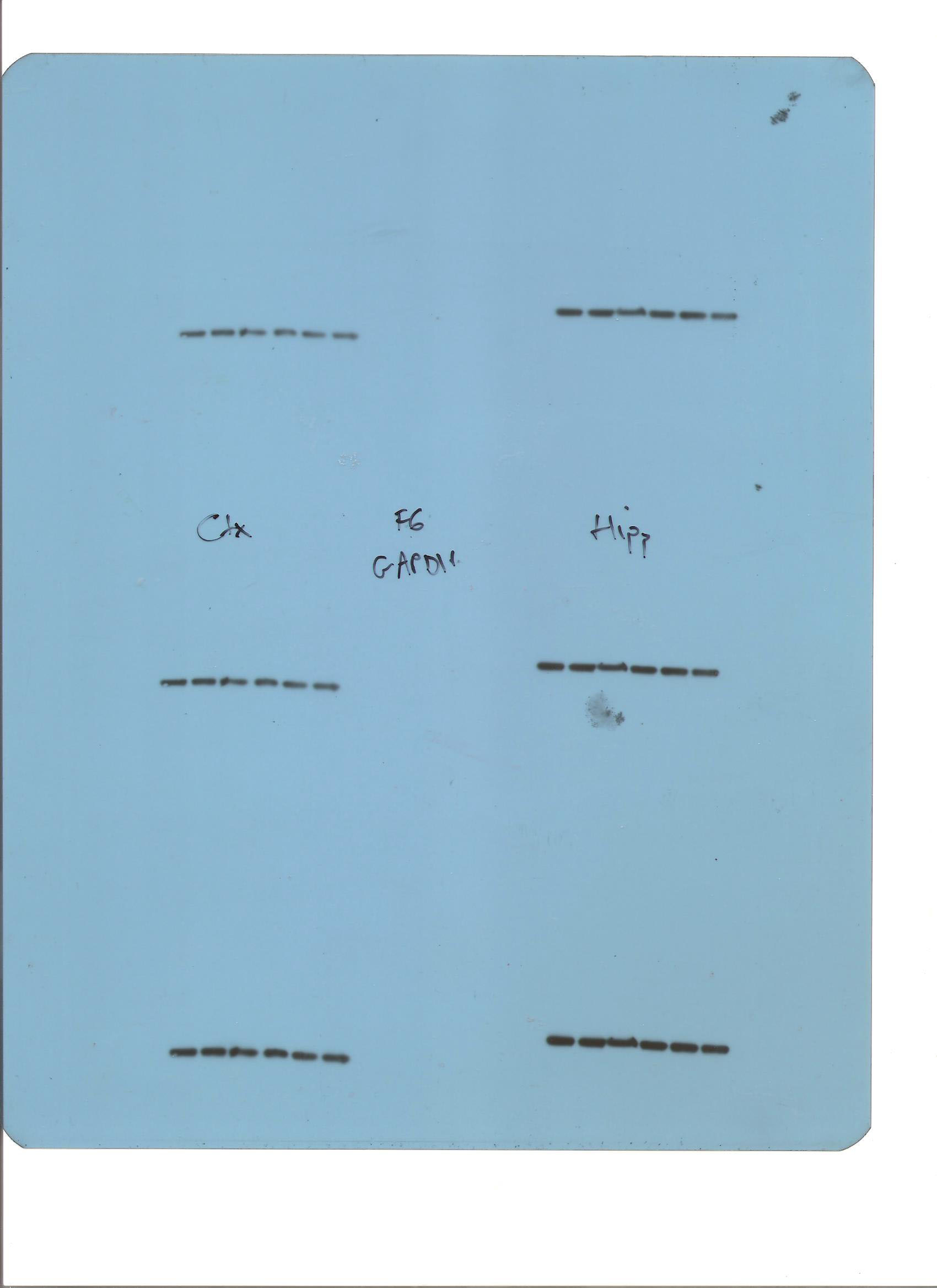

Supplement: Supplementary file 7 — Source data Fig. 6 [file 44321_2024_110_MOESM7_ESM.zip › 6B/6A n2 ctx and hipp GAPDH BRD0320.jpg]

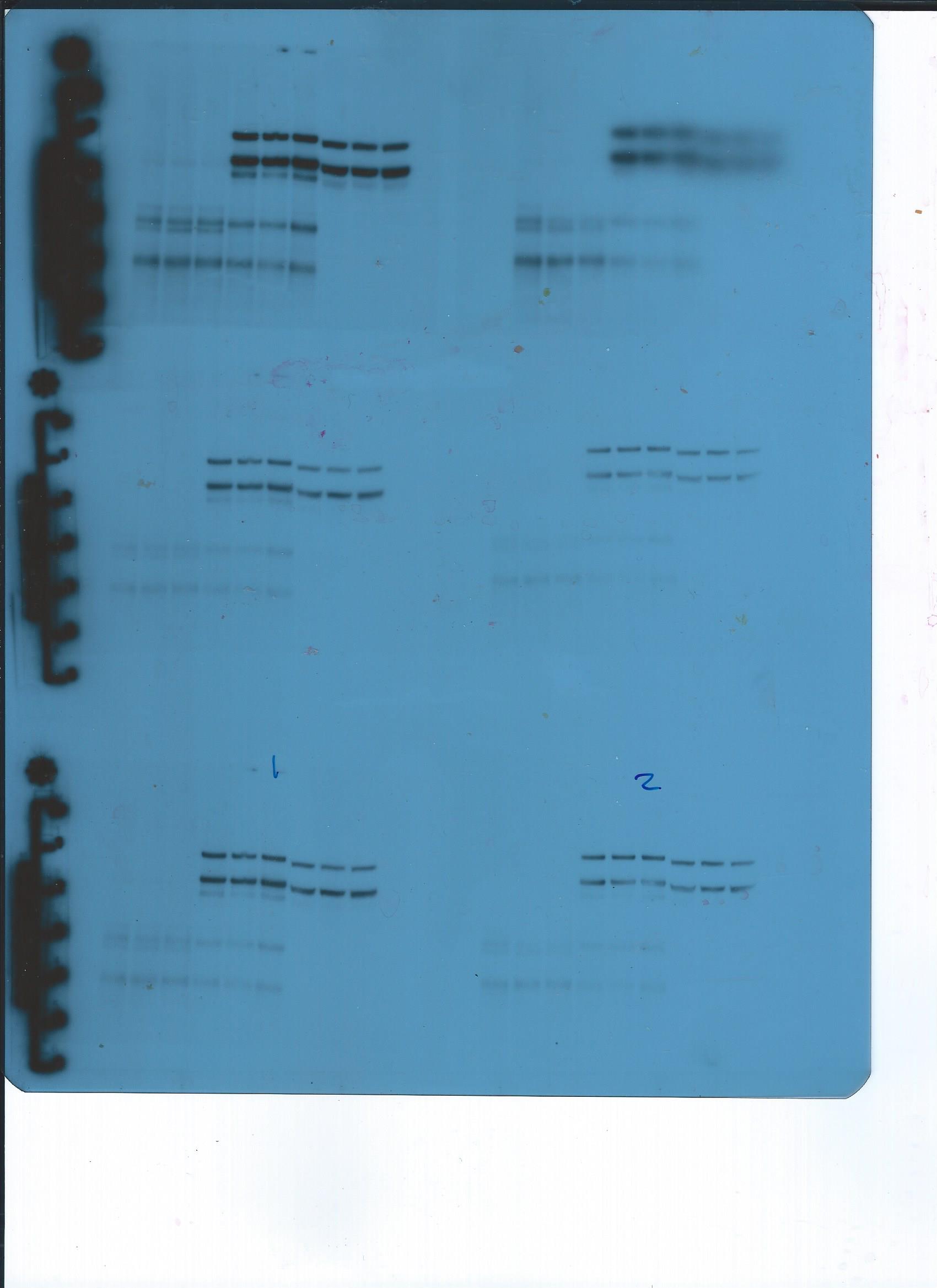

Supplement: Supplementary file 7 — Source data Fig. 6 [file 44321_2024_110_MOESM7_ESM.zip › 6C/6B N-cad IP BRD0320 set 1-2 .jpg]

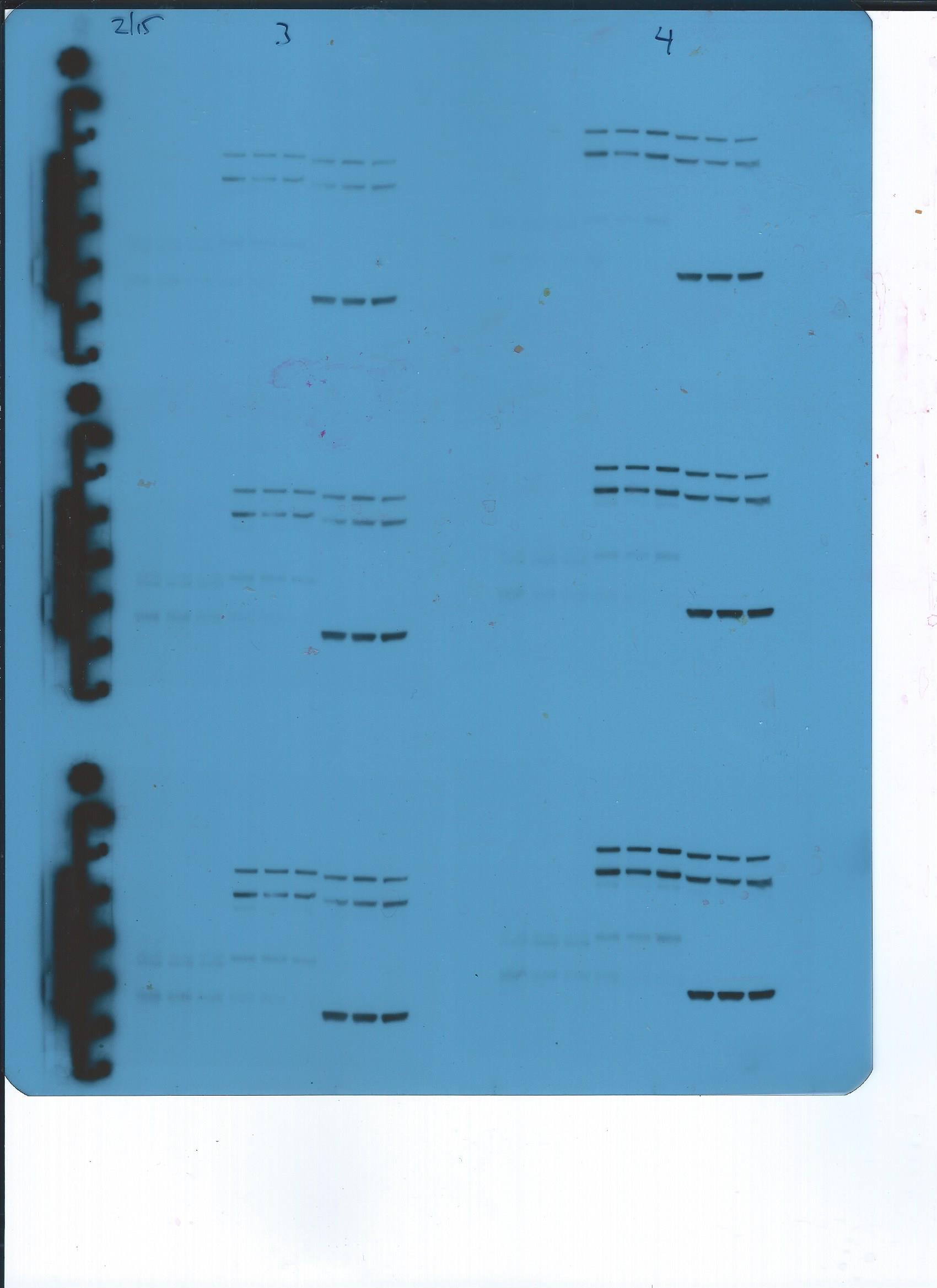

Supplement: Supplementary file 7 — Source data Fig. 6 [file 44321_2024_110_MOESM7_ESM.zip › 6C/6B N-cad IP BRD0320 set 3-4 .jpg]

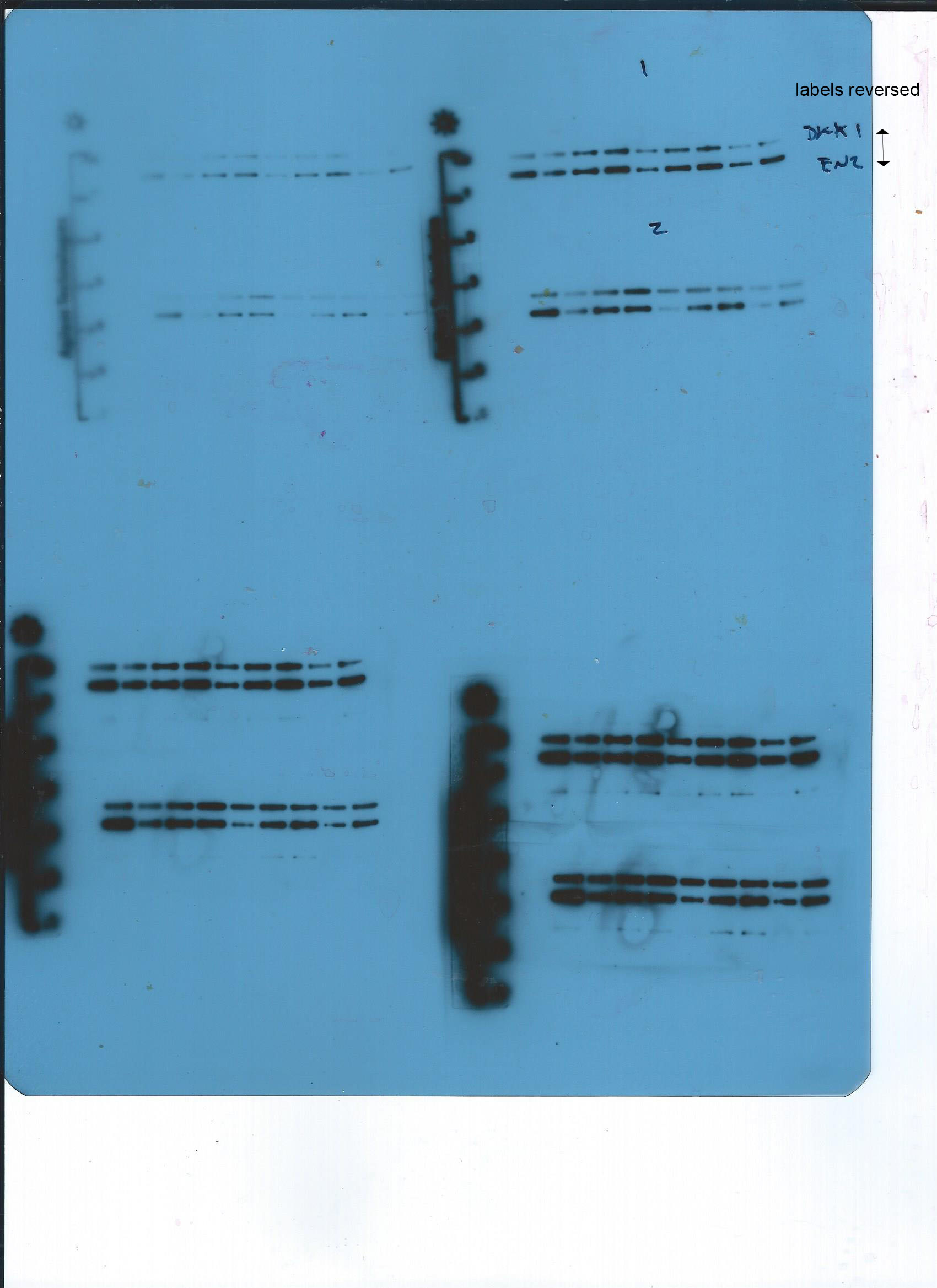

Supplement: Supplementary file 7 — Source data Fig. 6 [file 44321_2024_110_MOESM7_ESM.zip › 6D/6C DKK1 EN2 BRD0320.jpg]

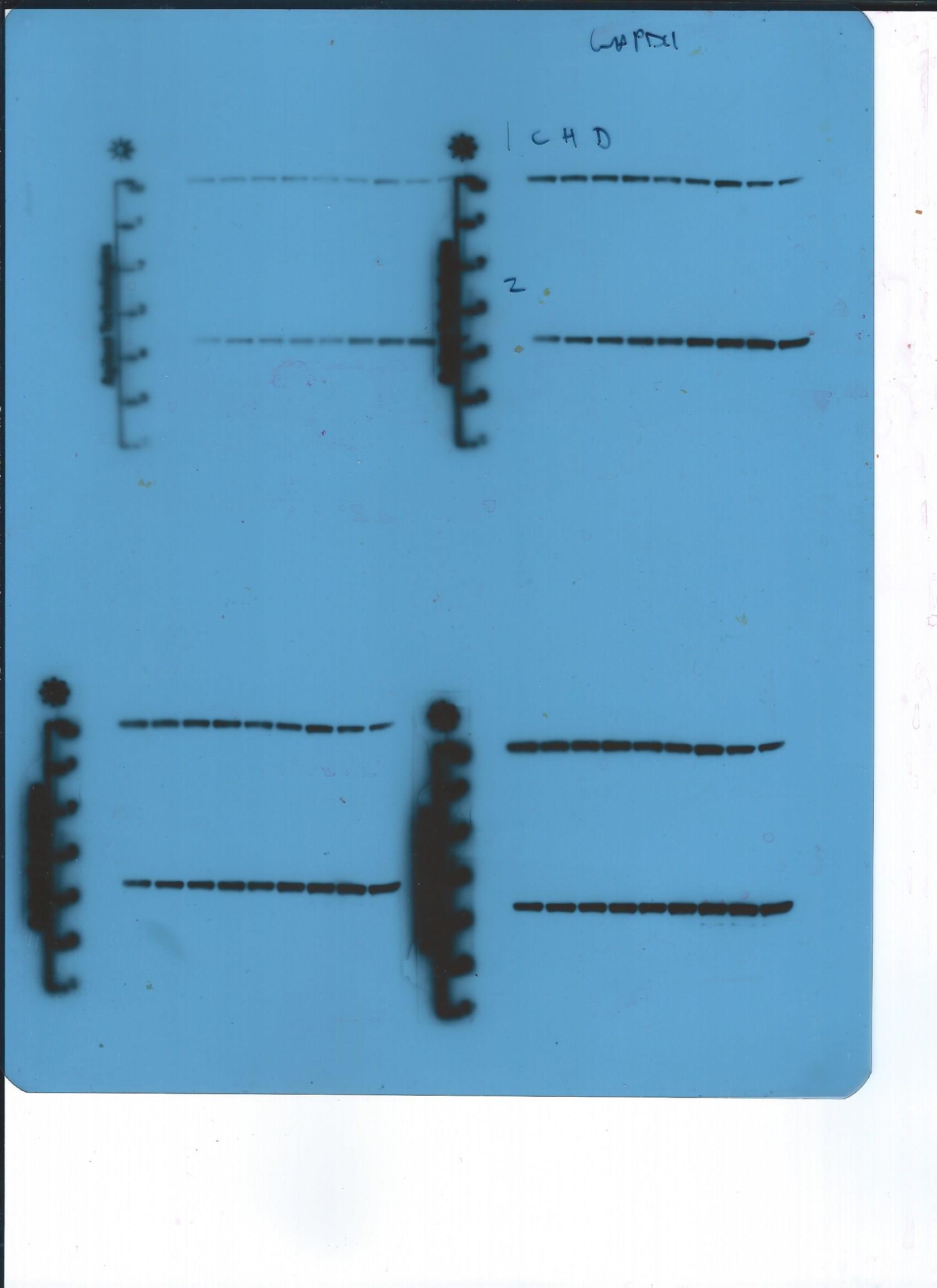

Supplement: Supplementary file 7 — Source data Fig. 6 [file 44321_2024_110_MOESM7_ESM.zip › 6D/6C GAPDH.jpg]
