# Supplementary material for: Inhibition of GSK3α,β rescues cognitive phenotypes in a preclinical mouse model of CTNNB1 syndrome
Source: EMBO Mol Med. 2024 Aug 5;16(9):8. doi: 10.1038/s44321-024-00110-5 (PMC11393422; doi:10.1038/s44321-024-00110-5)
Supplement: Supplementary file 11 — Expanded View Figures [file 44321_2024_110_MOESM11_ESM.pdf]

Expanded View Figures

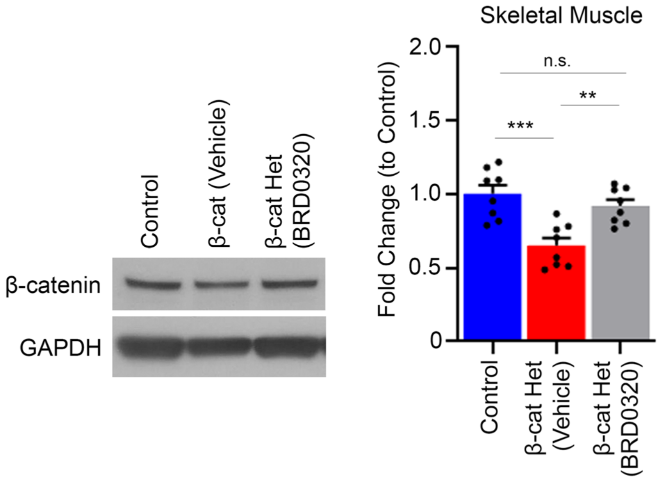

Figure EV1. Reduced β-catenin levels in skeletal muscle of β-cat mice are normalized by dual inhibition of GSK3α,β.

Immunoblots and quantification show reduced protein levels of β-catenin in skeletal muscle (diaphragm) of β-cat het mice that can are increased to levels not significantly different from wild-type littermates by treatment with GSK3α,β dual inhibitor ($n = 8$ per genotype/condition; one-way ANOVA $F[2, 21] = 12.85$, $P < 0.001$; Tukey's multiple comparison *t* test Control vs. vehicle-treated β-cat het, ***$P < 0.001$, Control vs. β-cat het dual inhibitor, **$P = 0.004$, β-cat het vehicle vs. β-cat het dual inhibitor, $P = 0.507$). All values are reported as mean of biological replicates +/− s.e.m. from two independent experiments. Source data are available online for this figure.

 © The Author(s)

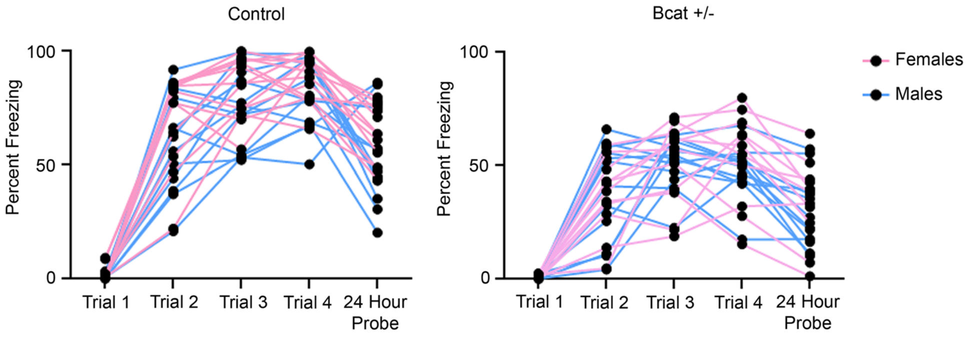

Figure EV2. Individual mouse freezing behavior in the contextual fear conditioning task plotted across all training and the 24-hour probe trials show no single β-cat het mouse performed at consistently lowest levels and no sex difference in the cognitive capabilities.

Control mice- $n = 13$ females, 12 males; repeated-measures ANOVA $F[1,23] = 7.006$ $P = 0.014$, Bonferroni's corrected t test Trial 1: $P > 0.999$; Trial 2: $P = 0.531$; Trial 3: $P = 0.586$; Trial 4: $P = 0.736$; 24 h probe: $P = 0.443$); β-cat het mice- $n = 11$ females, 12 males; repeated-measures ANOVA $F[1,21] = 0.136$ $P = 0.716$, Bonferroni's corrected t test Trial 1: $P > 0.999$; Trial 2: $P > 0.999$; Trial 3: $P > 0.999$; Trial 4: $P > 0.999$; 24 h probe: $P > 0.999$). All values are reported as mean of biological replicates +/− s.e.m. from two independent experiments. Source data are available online for this figure.

© The Author(s)

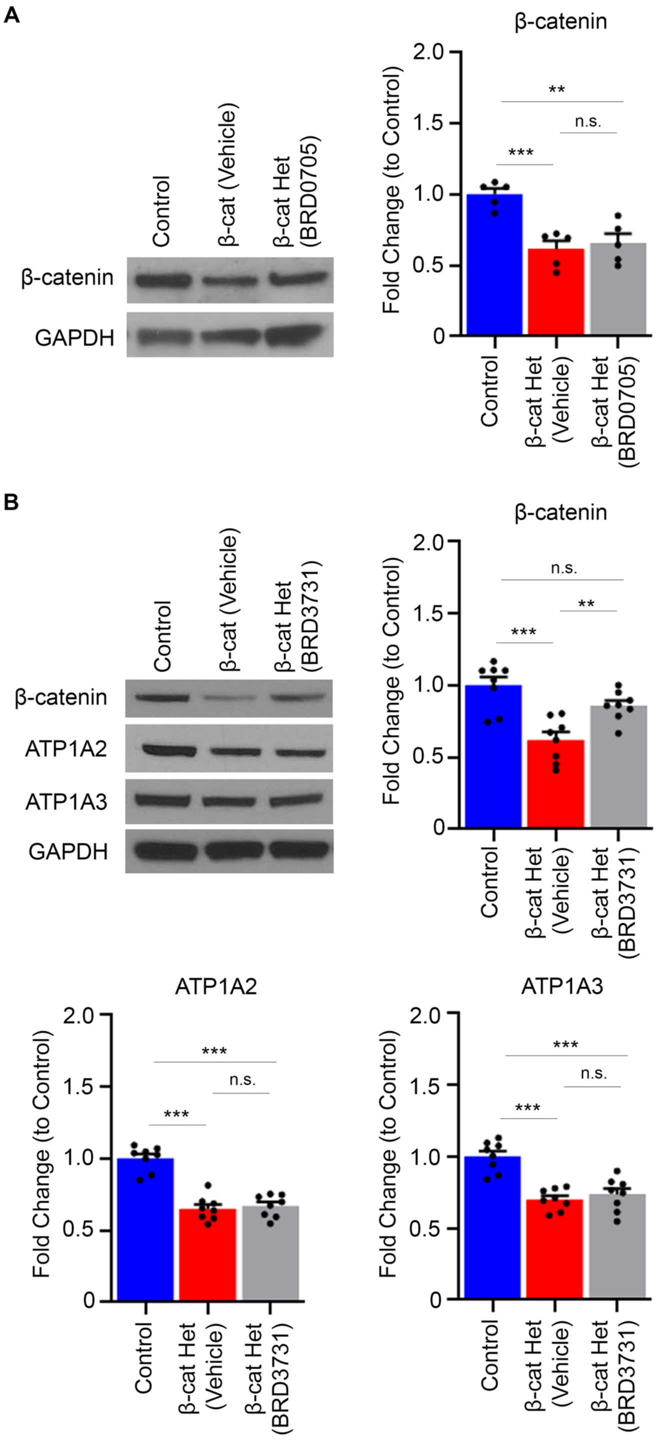

Figure EV3. GSK3α and GSK3β selective inhibitors do not rescue molecular changes to the same extent as inhibition of both isoforms.

(**A**) Immunoblots and quantification show that selective inhibition of the GSK3α isoform alone does not alter the reduced β-catenin protein levels in the β-cat het hippocampus, relative to vehicle-treated β-cat hets ($n = 5$ per genotype/condition; one-way ANOVA $F[2, 12] = 14.57$, $P < 0.001$; Tukey's multiple correction t test Control vs. vehicle-treated β-cat, ***$P < 0.001$, Control vs. β-cat het GSK3α inhibitor, **$P = 0.002$, β-cat het vehicle vs. β-cat het GSK3α inhibitor, $P = 0.856$). (**B**) Selective inhibition of the GSK3β isoform significantly increases β-catenin protein levels in the hippocampus, relative to vehicle-treated β-cat hets, wherease it fails to significantly increase the reduced levels of the Na/K ATPase α2 and α3 isoforms ($n = 8$ per genotype/condition; β-catenin: one-way ANOVA $F[2, 21] = 14.89$, $P < 0.001$; Tukey's multiple comparison t test Control vs. vehicle-treated β-cat het, ***$P < 0.001$, Control vs. β-cat het GSK3β inhibitor, $P = 0.127$, β-cat het vehicle vs. β-cat het GSK3β inhibitor, **$P = 0.008$; α2: one-way ANOVA $F[2, 21] = 44.78$, $P < 0.001$; Tukey's multiple comparison t test Control vs. vehicle-treated β-cat het, ***$P < 0.001$, Control vs. β-cat het GSK3β inhibitor, ***$P < 0.001$, β-cat het vehicle vs. β-cat het GSK3β inhibitor, $P = 0.898$; α3: one-way ANOVA $F[2, 21] = 21.35$, $P < 0.001$; Tukey's multiple comparison t test Control vs. vehicle-treated β-cat het, ***$P < 0.001$, Control vs. β-cat het GSK3β inhibitor, ***$P < 0.001$, β-cat het vehicle vs. β-cat het GSK3β inhibitor, $P = 0.776$). All values are reported as mean of biological replicates +/− s.e.m. from two independent experiments. Source data are available online for this figure.

 © The Author(s)

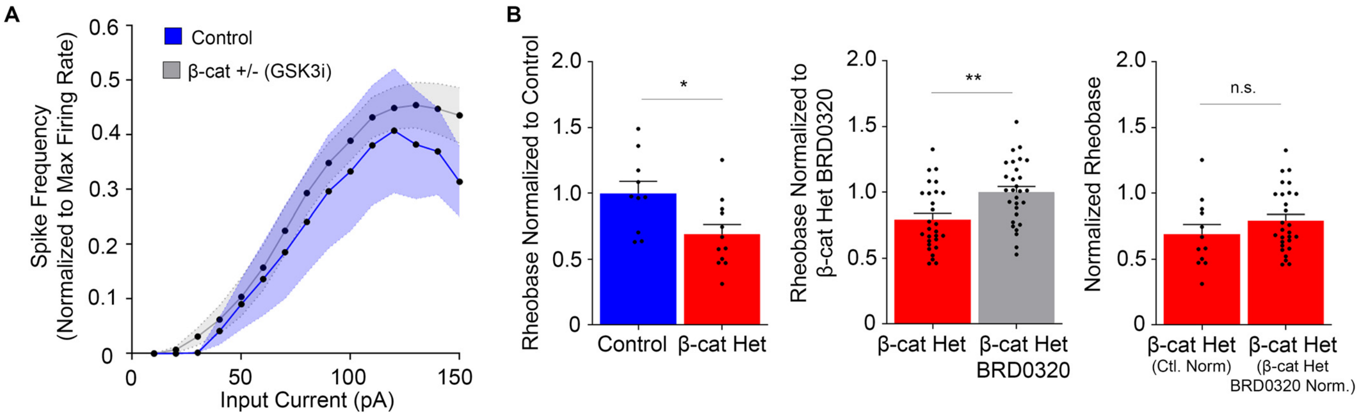

Figure EV4. GSK3α,β dual inhibitor improves excitability of β-cat het mice to resemble control levels.

(**A**) β-cat het mice treated with GSK3α,β dual inhibitor display spike frequency not significantly different from control littermates ($n = 10$ Controls, $n = 29$ β-cat het; repeated-measures ANOVA $F[1, 37] = 0.8189$, $P = 0.371$). (**B**) Histogram comparisons of the improved rheobase of β-cat hets treated with GSK3 dual inhibitor relative to vehicle-treated hets and wild-type littermates. Rheobases were normalized to either control (left panel: $n = 10$ Control, $n = 12$ β-cat het; Student's t test $P = 0.016$) or GSK3α,β inhibitor-treated β-cat hets (center panel: $n = 28$ β-cat het vehicle-treated, $n = 29$ β-cat het GSK3i-treated; Student's t test $P = 0.002$). Vehicle-treated β-cat hets showed significant decreases, to a similar extent, between the two normalizations suggesting no significant difference in excitability of GSK3 dual inhibitor hets compared to wild-types (right panel: $n = 12$ β-cat het normalized to Control, $n = 28$ β-cat het normalized to GSK3i-treated β-cat het; Student's t test $P = 0.224$). All values are reported as mean of biological replicates +/− s.e.m. from two independent experiments. Source data are available online for this figure.

© The Author(s)

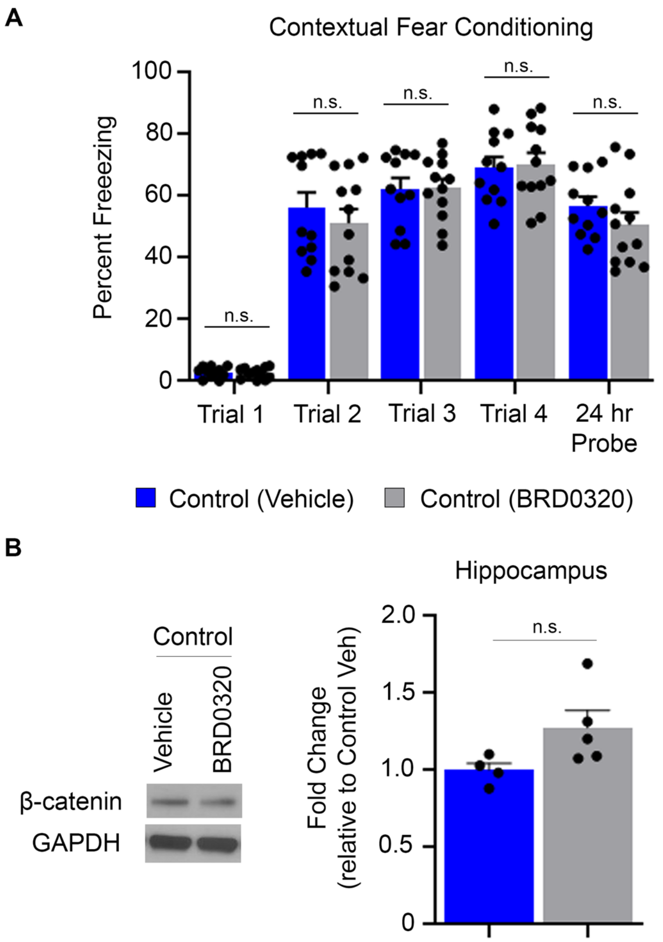

Figure EV5. GSK3α,β dual-paralog inhibitor does not alter learning or β-catenin levels in wild-type mice.

(**A**) Dual-paralog inhibition of GSK3α,β in wild-type mice does not significantly change their freezing behavior in the training trials and 24 h probe test in the contextual fear conditioning task, compared to vehicle-treated wild-type mice ($n = 11$ Controls vehicle-treated, 12 Controls dual inhibitor-treated; repeated-measures ANOVA $F[1,21] = 0.7150$, $P = 0.407$). (**B**) Further, it does not significantly alter β-cat protein levels in the hippocampus ($n = 4$ Controls vehicle-treated, 5 Controls dual inhibitor-treated; Student's t test, $P = 0.578$). All values are reported as mean of biological replicates $+/-$ s.e.m. from two independent experiments. Source data are available online for this figure.

 © The Author(s)

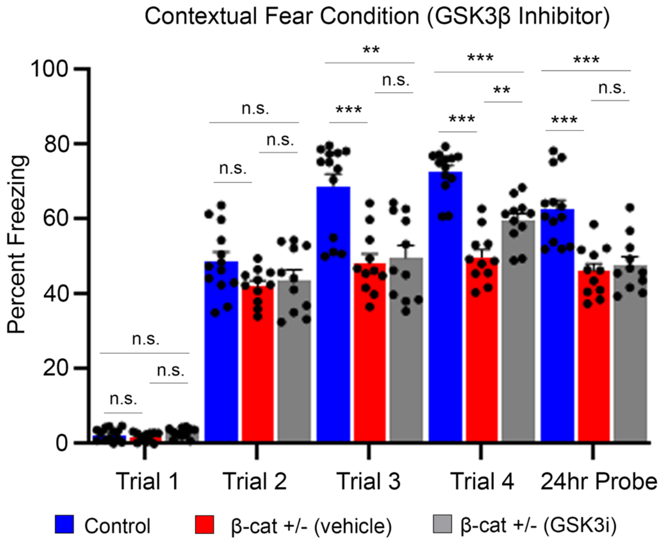

Figure EV6. GSK3β selective inhibitor does not correct cognitive deficits of β-cat het mice to the same extent as the GSK3α,β dual inhibitor.

Inhibition of GSK3β alone shows a modest significant improvement in freezing behavior at trial 4 that is not retained in the 24 h probe trial ($n = 13$ Controls, $n = 11$ vehicle-treated β-cat het, $n = 11$ GSK3β inhibitor-treated β-cat het; repeated-measures ANOVA $F[2, 32] = 73.32$, $P < 0.001$; Tukey's multiple comparison t test Trial 1: Control vs. β-cat het vehicle, $P = 0.586$; Control vs. β-cat het GSK3β inhibitor, $P = 0.671$, β-cat het vehicle vs. β-cat het GSK3β inhibitor, $P = 0.122$; Trial 2: Control vs. β-cat het vehicle, $P = 0.085$; Control vs. β-cat het GSK3β inhibitor, $P = 0.384$, β-cat het vehicle vs. β-cat het GSK3β inhibitor, $P = 0.843$; Trial 3: Control vs. β-cat het vehicle, ***$P < 0.001$; Control vs. β-cat het GSK3β inhibitor, **$P = 0.002$, β-cat het vehicle vs. β-cat het GSK3β inhibitor, $P = 9.37$; Trial 4: Control vs. β-cat het vehicle, ***$P < 0.001$; Control vs. β-cat het GSK3β inhibitor, *$P < 0.001$, β-cat het vehicle vs. β-cat het GSK3β inhibitor, **$P = 0.006$; 24 h Probe: Control vs. β-cat het vehicle, ***$P < 0.001$; Control vs. β-cat het GSK3β inhibitor, ***$P < 0.001$, β-cat het vehicle vs. β-cat het GSK3β inhibitor, $P = 0.844$). All values are reported as mean of biological replicates +/− s.e.m. from two independent experiments. Source data are available online for this figure.

© The Author(s)
